# Azelaic Acid Induces Mitochondrial Biogenesis in Skeletal Muscle by Activation of Olfactory Receptor 544

**DOI:** 10.3389/fphys.2020.00329

**Published:** 2020-04-17

**Authors:** Trung Thanh Thach, Chunyan Wu, Kwang Yeon Hwang, Sung-Joon Lee

**Affiliations:** ^1^Department of Biotechnology, School of Life Sciences and Biotechnology for BK21-PLUS, Korea University, Seoul, South Korea; ^2^Department of Food Bioscience and Technology, College of Life Sciences and Biotechnology, Korea University, Seoul, South Korea; ^3^Division of Biotechnology, College of Life Sciences and Biotechnology, Korea University, Seoul, South Korea

**Keywords:** azelaic acid, olfactory receptor 544, skeletal muscle, mitochondrial biogenesis, myotube

## Abstract

Mouse olfactory receptor 544 (Olfr544) is ectopically expressed in varied extra-nasal organs with tissue specific functions. Here, we investigated the functionality of Olfr544 in skeletal muscle cells and tissue. The expression of Olfr544 is confirmed by RT-PCR and qPCR in skeletal muscle cells and mouse skeletal muscle assessed by RT-PCR and qPCR. Olfr544 activation by its ligand, azelaic acid (AzA, 50 μM), induced mitochondrial biogenesis and autophagy in cultured skeletal myotubes by induction of cyclic adenosine monophosphate-response element binding protein (CREB)-peroxisome proliferator-activated receptor gamma coactivator 1-alpha (PGC-1α)-extracellular signal-regulated kinase-1/2 (ERK1/2) signaling axis. The silencing Olfr544 gene expression abrogated these effects of AzA in cultured myotubes. Similarly, in mice, the acute subcutaneous injection of AzA induced the CREB-PGC-1α-ERK1/2 pathways in mouse skeletal muscle, but these activations were negated in those of Olfr544 knockout mice. These demonstrate that the induction of mitochondrial biogenesis in skeletal muscle by AzA is Olfr544-dependent. Oral administration of AzA to high-fat-diet fed obese mice for 6 weeks increased mitochondrial DNA content in the skeletal muscle as well. Collectively, these findings demonstrate that Olfr544 activation by AzA regulates mitochondrial biogenesis in skeletal muscle. Intake of AzA or food containing AzA may help to improve skeletal muscle function.

## Introduction

Olfactory receptors (ORs) are G-protein coupled receptors (GPCR), which are mainly expressed in the cilia of the olfactory epithelium (Buck and Axel, 1991). Binding of a ligand odorant stimulates signal transduction pathways to transduce odor information to the brain (Firestein, 2001). It has also been reported that ORs are ectopically expressed in different extra-nasal tissues, including liver, kidney, adipose, intestine and muscle tissues (Lee et al., 2019). Thus, the functionalities of ectopic ORs have been investigated in the last decade. For instance, MOR23 activation stimulates the cyclic adenosine monophosphate (cAMP) levels and protein kinase A (PKA) activity in skeletal muscle tissue. This pathway regulates the migration and adhesion of skeletal muscle cells, thereby contributing to wound healing and tissue repair (Griffin et al., 2009). A few ORs regulate lipid metabolism and obesity. We previously reported that OR1A1 and its mouse homolog, Olfr43, regulate lipid metabolism in the liver. OR1A1/Olfr43 stimulated by (-)-carvone reduced hepatic steatosis through regulating the PKA-cAMP-response element binding protein (CREB)- hairy and enhancer of split-1 signaling axis (Wu et al., 2015, 2019). The results from microarray analysis showed that Olfr544 is highly expressed in both mouse liver and white adipose tissue, and activation of Olfr544 stimulates fatty acid oxidation in hepatocytes, lipolysis and thermogenesis in white and brown adipose tissues (Wu et al., 2017), respectively. Olfr544 is also expressed in pancreatic α-cells to stimulate glucagon secretion (Kang et al., 2015). These results suggest that ectopic ORs expressed in non-nasal tissues can play a role in functional GPCR proteins and stimulate unique signal transduction pathways, resulting in tissue-specific roles by recognizing odorants as ligand molecules.

Skeletal muscle is a major organ of ATP consumption, which is critical for sustaining oxidative metabolism and homeostasis of the ATP pool in healthy individuals (Russell et al., 2014). Under intensive exercise, nearly 90% of cardiac output is distributed to skeletal muscle. Regulating the energy metabolism of skeletal muscle is critical to maintain normal physiology. It has been shown that energy metabolism of the skeletal muscle is largely regulated by mitochondrial function and a balance between mitochondrial biogenesis and the autophagy pathway (Russell et al., 2014). Enrichment of mitochondria in skeletal muscle improves oxygen uptake capacity and reduces adipose tissue mass, thus increasing exercise capacity and lowering the risk of type 2 diabetes and cardiovascular disease (Little and Cochran, 2011; Duclos et al., 2013; Russell et al., 2014).

Mitochondrial contents in skeletal muscle can be stimulated by mitochondrial biogenesis (Yan et al., 2012; Perry and Hawley, 2018), which is regulated by multiple signaling pathways, including peroxisome proliferator-activated receptor-γ coactivator 1α (PGC-1α). PGC-1α is stimulated by several kinases, including CREB and extracellular signal-regulated protein kinases 1/2 (ERK1/2). PGC-1α is also activated through deacetylation by the NAD-dependent protein deacetylase sirtuin-1 (SIRT1) (Gerhart-Hines et al., 2007; Wright et al., 2007; McConell et al., 2010). PGC-1α activation induces downstream transcription factors, such as nuclear respiratory factors (NRF1 and NRF2) and mitochondrial transcription factor A (TFAM), which upregulate genes encoding mitochondrial biogenesis and electron transport chain proteins (Wu et al., 1999; Russell et al., 2014). Thus, PGC-1α is well involved in mitochondrial biogenesis and function (Schmidt and Mandrup, 2011; Scarpulla et al., 2012).

AzA is a C9 α,ω-dicarboxylic acid (nonanedioic acid) that is found in grain foods, including oatmeal and barley (Gallagher et al., 2010), and is also endogenously produced by the peroxisomal ω-oxidation pathway as an end product of linoleic acid (Litvinov et al., 2010). AzA is a ligand for the mouse olfactory receptor Olfr544 (Kang et al., 2015; Wu et al., 2017); thus, oral administration of AzA in mice reduces adiposity, rewiring fuel preference to fats (Wu et al., 2017). Our microarray analysis of mouse skeletal muscle tissues identified Olfr544 as the most highly expressed OR. Therefore, we further investigated the biological function of AzA on mitochondrial biogenesis in skeletal muscle cells both *in vitro* and *in vivo*. Moreover, the molecular mechanism of Olfr544-mediated mitochondrial biogenesis in the muscle was also examined in both wild-type and Olfr544-deficient mice.

## Materials and Methods

### Cell Culture, Differentiation, and Compound Treatment

The C2C12 cells (American Type Culture Collection, United States) were cultured in Dulbecco’s modified Eagle medium (DMEM, Gibco, MA, United States) containing with 20% fetal bovine serum (FBS, HyClone, IL, United States), 100 units/mL of penicillin and 100 mg/mL streptomycin (PEST, Sigma-Aldrich, St. Louis, MO, United States) at 37°C with 5% CO_2_ (v/v). The cells were differentiated as previously described (Thach et al., 2016). Briefly, mouse skeletal muscle C2C12 cells were switched to DMEM containing 2% horse serum (HyClone). After a 7-day differentiation, cells were treated with AzA (Sigma) in serum-free DMEM for 24 h. DMSO (0.1%, Bio Basic Canada Inc., Canada) was used as a control.

### Double-Transfection of Small Interfering RNA (siRNA)

C2C12 cells were seeded overnight and differentiated for 7 days. Differentiated skeletal myotubes were transfected with 200 pmol of scramble or *Olfr544* siRNA duplex (SantaCruz, CA, United States) with Lipofectamine 2000 reagent (Invitrogen, CA, United States) as previously described (Wu et al., 2019). After transfection for 6 h, differentiated skeletal myotubes were transfected again with the same amount of scramble or *Olfr544* siRNA. After 5 h of double transfection, cells were added with fresh DMEM containing 20% FBS. Subsequently, transfected cells were treated for 10h with DMSO or AzA before total mRNA or protein extraction.

### Quantitative Real-Time RT-PCR

The reagent of RNAiso Plus (TaKaRa Bio Inc., Otsu, Japan) was used to extract the total RNA of C2C12 cells and muscle tissues. Subsequently, Rever Trace RT Master Mix Kit (Toyobo, Osaka, Japan) was used to synthesize the cDNA according to the manufacturer’s instructions using the. Quantitative RT-PCR experiments were then conducted to check the gene expression levels with cDNA as previously described (Jia et al., 2013; Kang et al., 2015; Wu et al., 2019). Templates were amplified by using specific sets of primers listed in Supplementary Table S1 with the Thunderbird^TM^ SYBR qPCR Mix reagent (Takara Bio Inc., Japan) and analyzed by the iQ5 Cycler System (Bio-Rad, Hercules, CA, United States). *Olfr544* mRNA levels was quantified in reference to pME18S-Olfr544 plasmid and normalized to ribosomal protein L32 levels.

### Immunoblotting Analysis

Immunoblotting analysis was used to measure the protein levels of C2C12 and muscle tissues (Jun et al., 2014; Hoang et al., 2015; Jia et al., 2016). Briefly, lysates of skeletal muscle cells and tissues were obtained in a radioimmunoprecipitation assay buffer containing protease and phosphatase inhibitors (Thermo, Waltham, MA, United States). The protein levels were checked using protein assay dye reagent (Bio-Rad, Hercules, CA, United States). Subsequently, SDS-PAGE were used to separate the denatured proteins. The separated proteins were then transferred to the nitrocellulose membranes (Daeillab, Seoul, South Korea). The membranes were incubated overnight with primary antibodies at 4°C. Antibodies for CREB (1:250), p-CREB (Ser133; 1:500), β-actin (1:1000), α-tubulin (1:1000), ERK1/2 (1:500), p-ERK1/2 (Thr53/54, 1:500), PGC-1α (1:500) were purchased from Santa Cruz Biotechnology (United States); anti-LC3B (1:500) from Novus Biologicals (Novus Biologicals, Littleton, CO, United States). Immunoblotting images were accessed by a ChemiDoc^TM^ touch imaging system, and analyzed by the Image Lab 5.2 software (Bio-Rad, PA, United States). The protein levels of α-tubulin or β-actin were used for normalization.

### Mitochondrial DNA Content and Abundance Determination

Mitochondrial DNA content and abundance were determined as previously described (Thach et al., 2016). Mitotracker Green probe (Molecular Probes) was used to measure the mitochondrial density following the manufacturer’s instructions. Briefly, C2C12 cells were stained with Green probes (200 nm) for 30 min at 37°C after washing with PBS (pH 7.4). Subsequently, the green fluorescence intensity was measured using SpectraMAX (Molecular Devices Co.), at the wavelength of 490 nm (excitation) and 516 nm (emission), respectively. The images were obtained by the Zeiss LSM700 confocal microscope, and then analyzed using the Zeiss LSM700 version 3.2 software (Carl Zeiss, Germany).

### Mouse Care and Experiments

Healthy, male, 8-week-old ICR, and C57BL/6J mice weighing 20–25 g were purchased from Samtako (Gyeonggi-do, South Korea). Generations of Olfr544 knockout mice were generated using the CRISPR/Cas9 system to delete exon 2 (161–428 bp) of the O*lfr*544 gene, and the method and basic characteristics of Olfr544 knockout mice (KO) were previously published (Wu et al., 2017). Animal experiments were handled in accordance with the protocols approved by the Animal Experiment Committee of Korea University (Protocol No. KUIACUC-2019-0031). Animals were kept in the animal room with a 12 h photoperiod and a relative humidity of 50–60% at 21–25°C. Mice were allowed free access to 60% high fat diet (HFD) and randomly assigned into four groups (*n* = 7), two groups each for wild-type and Olfr544 knockout mice. For acute Olfr544 activation, mice were fasted overnight and intraperitoneally injected with either AzA (100 mg/kg body weight) or PBS (vehicle group). Skeletal muscle tissues (soleus muscles) were collected at indicated time as previously described (Jia et al., 2015). For long-term AzA administration, mice were orally administered either AzA (50 mg/kg body weight) or ddH_2_O under HFD. The body weights of mice were recorded every week. After oral feeding for 6 weeks, mice were anesthetized and sacrificed after overnight fasting. Muscle tissues were collected, immediately cryoprotected, and then stored at –80°C for further experiments.

### Statistical Analysis

The data are shown as the means ± SEM. To determine significance between two or multiple groups, Wilcoxon test and one-way ANOVA followed by Tukey’s HSD test were used, respectively. Data are statistically significant different denoted by ^∗^ for *P* ≤ 0.05, ^∗∗^ for *P* ≤ 0.01.

## Results

### Olfr544 Is Expressed in Cultured C2C12 Derived Myotubes, and Its Activation Induces the PKA-CREB-PGC-1α Signaling Axis

In a microarray analysis of mice fed normal CHOW and high-fat diet (HFD), Olfr544 was the most highly expressed OR in skeletal muscles. The expression levels of Olfr544 were not significantly changed by HFD (Supplementary Methods and Supplementary Figure S1). The expression of Olfr544 was further confirmed in differentiated C2C12 myotubes and mouse skeletal muscle tissues using RT-PCR (Supplementary Figure S2A). Olfr545, which shares 95% sequence homology with Olfr544, was also expressed at low levels, with approximately 20% of Olfr544 expression (Supplementary Figure S2A). In the CRE-luciferase reporter gene assay, AzA, a ligand of Olfr544, weakly activated Olfr545; thus, the EC50 value of AzA for Olfr545 was 12-fold greater than that of Olfr544 (EC50; 19.2 ± 4.8 vs. 237 ± 140 μM for Olfr544 and Olfr545, respectively, Supplementary Figures S2B,C). Further experiments were performed with AzA concentrations to selectively stimulate Olfr544 but not Olfr545.

**FIGURE 1:**
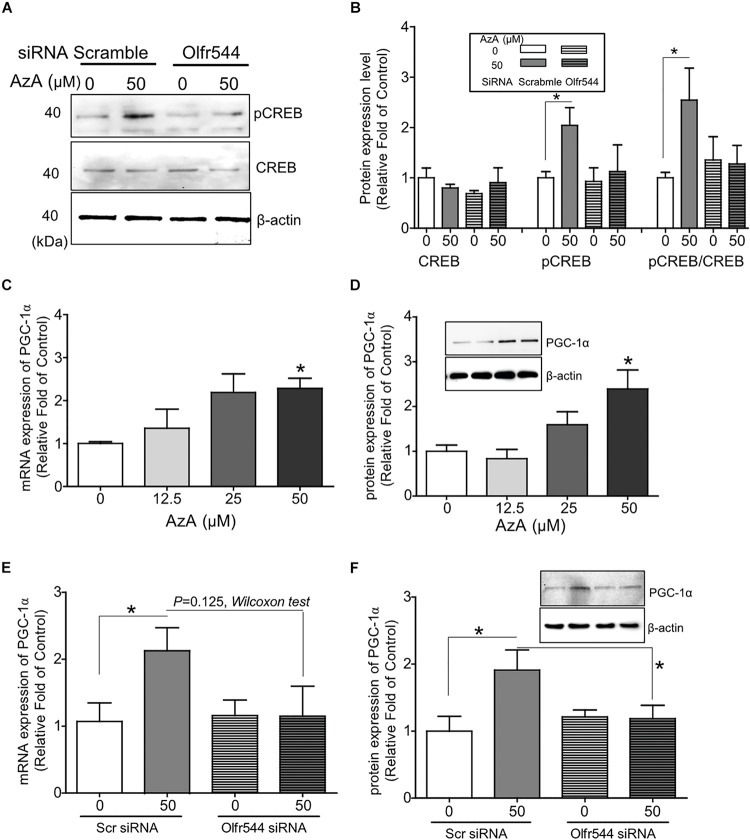
Activation of Olfr544 induces the PKA-CREB-PGC-1α signaling axis in cultured skeletal muscle cells. **(A,B)** AzA induced pCREB expression in C2C12 myotubes but not in cells with Olfr544 knockdown. Immunoblotting analysis of pCREB and total CREB proteins (A, *n* = 3); the ratios of pCREB-to-CREB were normalized to β-actin (**B**, *n* = 3). **(C,D)** AzA induced the expression of PGC-1α both at the mRNA (**C**, *n* = 3) and protein levels (**D**, *n* = 3) in a dose-dependent manner as measured by real-time qPCR and immunoblotting, respectively. **(E,F)** Olfr544 gene knockdown lessens Pgc-1α gene expression (**E**, *n* = 3) and protein expression (**F**, *n* = 3). Data are the mean ± SEM. Data are statistically significant different denoted by * for *P* ≤ 0.05 using Wilcoxon test and one-way ANOVA followed by Tukey’s HSD test.

In cultured C2C12 myotubes, AzA stimulated the PKA-CREB signaling axis (Supplementary Figure S2B), in line with the results from the CRE-luciferase assay (Supplementary Figure S2C). AzA induced pCREB levels by 1.5-fold in C2C12 myotubes; meanwhile, the induction of pCREB was abrogated in cells with Olfr544 knockdown (Figures 1A,B). The expression of Olfr544 in cultured Olfr544 knockdown myotubes was silenced by 80% with transfection of Olfr544 specific siRNA (Supplementary Figure S3). When differentiated C2C12 myotubes were stimulated with AzA (0–50 μM), the mRNA and protein expressions of PGC-1α in myotubes were induced a dose-dependent manner. AzA (50 μM) significantly induced the mRNA and protein expression levels of PGC-1α, by 2- and 3-fold, respectively (Figures 1C,D). However, these inductions were negated in Olfr544 knockdown cells (Figures 1E,F). We did not observe the significant difference of mRNA *Pgc-1*α expression stimulated by AzA in cells transfected with scramble or Olfr544 siRNA (*P* = 0.125). Nonetheless, we further confirmed that the PGC-1α protein expression was induced by AzA treatment in the presence of scramble siRNA and significantly higher the expression level in the presence of Olfr544 siRNA (Figure 1F). These data demonstrate that Olfr544 activation stimulates the CREB-PGC-1α signaling axis in cultured myotubes.

### AzA Induces Mitochondrial Biogenesis in Cultured C2C12 Derived Myotubes

**FIGURE 2:**
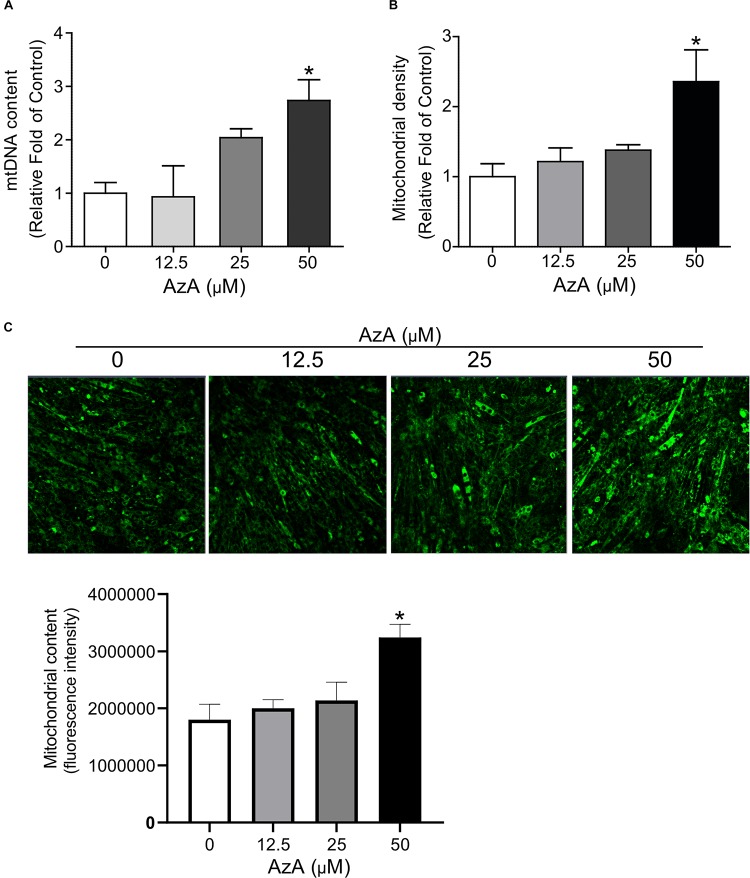
AzA induces mitochondrial biogenesis in skeletal muscle cells. **(A)** AzA induces mtDNA content in a dose-dependent manner as measured by Qpcr (*n* = 4). **(B)** Mitochondrial contents were probed by green MitoTracker and measured by a spectrophotometer (*n* = 4). **(C)** and the levels of mitochondrial content was confirmed under confocal fluorescence microscopy (*n* = 3). Blue, nucleus; green, mitochondrion. Scale bar, 50 μm. Data are the mean ± SEM. Data are statistically significant different denoted by * for *P* ≤ 0.05 using Wilcoxon test and one-way ANOVA followed by Tukey’s HSD test.

We next investigated whether AzA stimulates mitochondrial biogenesis in C2C12 derived myotubes since the CREB-PGC-1α signaling axis has been reported to activate mitochondrial biogenesis (Herzig et al., 2001; Schmidt and Mandrup, 2011). C2C12 cells were differentiated for 7 days and then treated with AzA for 24 h. Quantitative real-time PCR results showed that 50 μM AzA significantly increased the mtDNA content by 3.0-fold (Figure 2A). Similarly, mitochondrial density was significantly increased by approximately 2.5-fold (Figure 2B). Subsequently, MitoTracker-probed mitochondrial images observed under confocal fluorescence microscopy showed substantial increases in mitochondrial density (Figure 2C). These results suggest that AzA induces mitochondrial biogenesis in skeletal muscle cells.

### Olfr544 Gene Knockdown Negates Mitochondrial Biogenesis Stimulated by AzA in Cultured C2C12 Derived Myotubes

**FIGURE 3:**
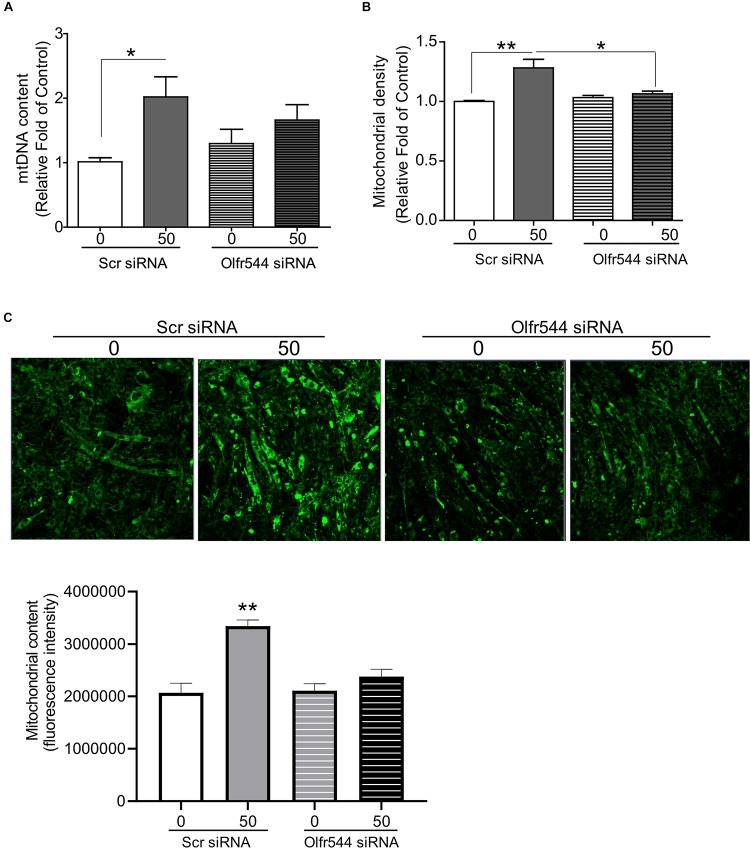
Olfr544 deficiency negates mitochondrial biogenesis stimulated by AzA in skeletal muscle cells. **(A)** AzA induced mtDNA content in myotubes but not in Olfr544 knockdown cells (*n* = 8). Mitochondrial abundance was analyzed using a spectrophotometer (**B**, *n* = 8) and fluorescence imaging (**C**, *n* = 3). Scale bar, 50 μm. Data are the mean ± SEM. Data are statistically significant different denoted by * for *P* ≤ 0.05, ** for *P* ≤ 0.01 using Wilcoxon test and one-way ANOVA followed by Tukey’s HSD test.

We next examined whether AzA regulates mitochondrial biogenesis via Olfr544 activation. C2C12 cells were transfected with scramble siRNA or Olfr544 siRNA after differentiation, and then treated with AzA for 24 h. Olfr544 knockdown negated the effect of AzA on mitochondrial biogenesis. The mtDNA content and mitochondria abundance were unaltered by AzA stimulation in Olfr544 knockdown cells (Figures 3A,B). In contrast, AzA-treated control C2C12 myotubes showed a significant induction of mtDNA content by 2.0-fold compared with controls. AzA treatment increased mtDNA content in normal cells by approximately 1.6-fold compared with Olfr544 knockdown cells (Figure 3A). Quantification of mitochondrial density showed the enrichment of mitochondria in cells stimulated with AzA (50 μM) in C2C12, but not in Olfr544 knockdown C2C12 myotubes (Figures 3B,C). However, the effects of AzA on mitochondrial biogenesis were impaired with siRNA transfection, which was smaller (Figure 3) than the effects in non-transfected cells (Figure 2). The results collectively demonstrate that AzA stimulates muscle mitochondrial function via Olfr544 activation.

### Olfr544 Activation Induces ERK1/2 Phosphorylation in Cultured C2C12 Derived Myotubes

**FIGURE 4:**
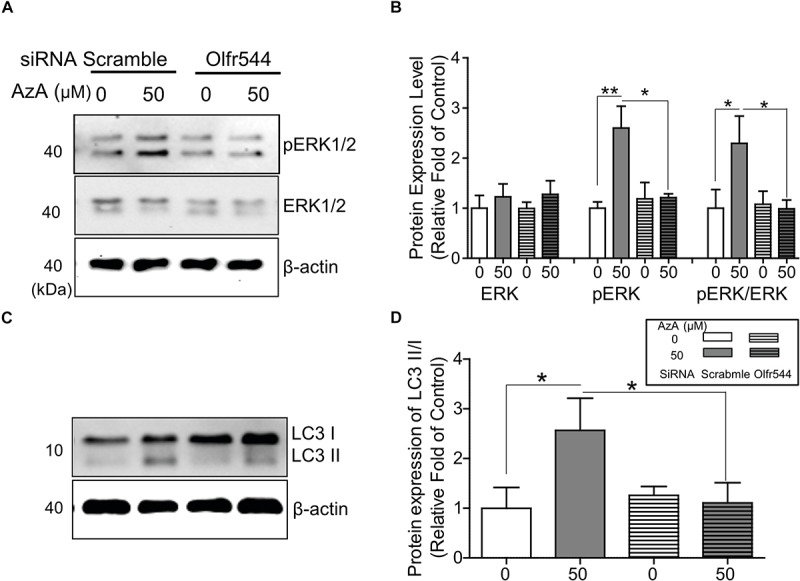
AzA-driven Olfr544 activation stimulates ERK1/2 phosphorylation in cultured skeletal muscle cells. AzA induced phosphorylation of ERK1/2 on Thr43/44 (pERK1/2) in myotubes but not in Olfr544 knockdown cells. **(A,B)** Expression of total ERK1/2 and pERK1/2 was probed by immunoblotting. Ratios of pERK1/2 to total ERK1/2 were normalized to β-actin (*n* = 3). **(C,D)** AzA-stimulated myotubes increased the LC3-II-to-LC3-I ratio, a marker of autophagosome formation, but not in Olfr544 knockdown cells (*n* = 3). Data are the mean ± SEM. Data are statistically significant different denoted by * for *P* ≤ 0.05, ** for *P* ≤ 0.01 using Wilcoxon test and one-way ANOVA followed by Tukey’s HSD test.

PGC-1α gene expression is alternatively induced by ERK1/2; thus, we next checked the phosphorylation level of ERK1/2 by AzA in differentiated C2C12 myotubes. In immunoblotting analysis, AzA significantly induced phosphorylations of ERK1/2 on Thr43/44 (pERK1/2) by 2.0-fold, but these effects were abrogated in Olfr544 knockdown cells (Figures 4A,B). It has been reported that increased pERK1/2 correlates to autophagy levels (Martinez-Lopez et al., 2013). Autophagy plays a pivotal role in skeletal muscle adaption and capacity by interacting with mitochondrial biogenesis and preventing mitochondrial damage (He et al., 2012; Lira et al., 2013; Lo Verso et al., 2014). In cultured C2C12 myotubes, activation of Olfr544 by AzA increased the LC3-II-to-LC3-I ratio, a marker of autophagosome formation, by 2.5-fold compared to vehicle-treated controls, while the induction disappeared in Olfr544 knockdown cells (Figures 4C,D). Importantly, AzA-stimulated cells showed an approximately 3.0-fold increase of the LC3-II-to-LC3-I ratio compared to the Olfr544 knockdown cells (Figure 4D). Taken together, Olfr544 activated by AzA increases ERK1/2 activity and induces autophagy formation in skeletal muscle cells.

### Olfr544 Activation Stimulates the CREB-PGC-1α Pathway and Autophagy Formation in Mouse Skeletal Muscle Tissues

**FIGURE 5:**
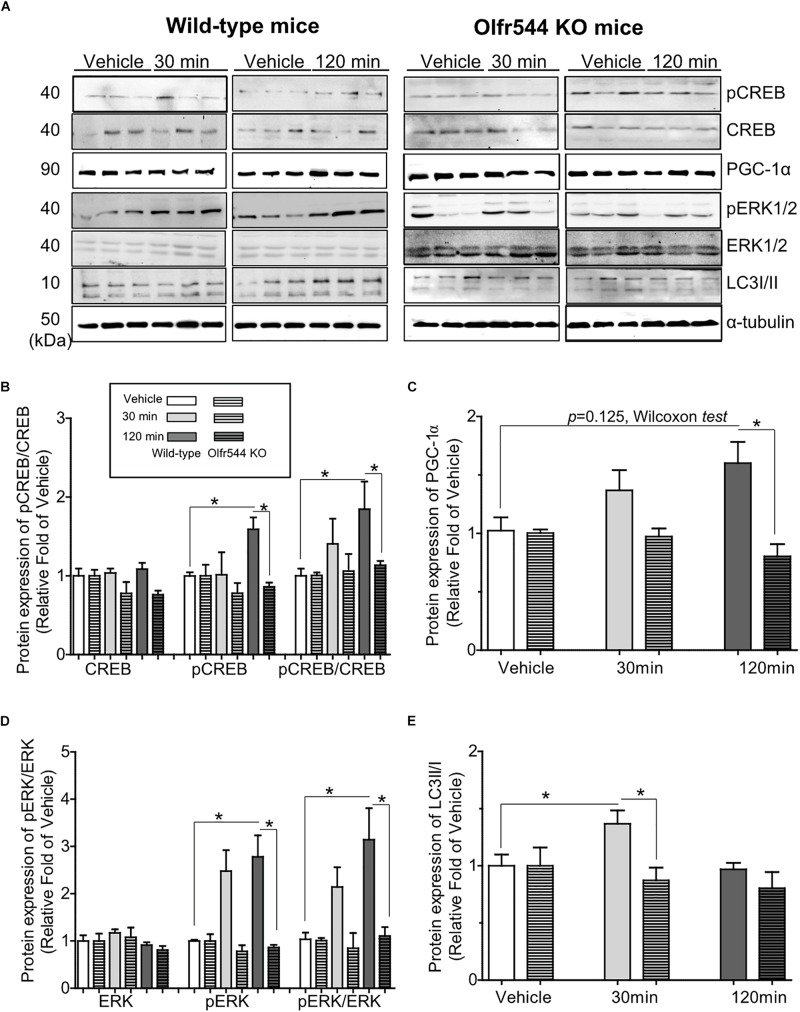
AzA-driven Olfr544 activation stimulates the CREB-PGC-1α signaling axis and autophagy formation in mouse skeletal muscle tissues. **(A)** AzA treatment induced the expression of PGC-1α, pCREB, pERK1/2 and LC3I/II protein in wild-type mouse skeletal muscle tissues but not in those of Olfr544 KO mice. Immunoblotting analysis of soleus muscles extracts for PGC-1α, pCREB, and CREB, pERK1/2 and total ERK1/2, LC3I/II (*n* = 3). **(B–E)** Ratios of pCREB, PGC-1α, pERK1/2, and LC3-II were normalized to β-actin (*n* = 3). Data are the mean ± SEM. Data are statistically significant different denoted by * for *P* ≤ 0.05 using Wilcoxon test and one-way ANOVA followed by Tukey’s HSD test.

We next investigated the biological activities of AzA in mouse skeletal muscle tissues *in vivo*. Mice were intraperitoneal injected with AzA (100 mg/kg body weight) for 30 or 120 min before soleus muscles collection for immunoblot analysis. Vehicle group mice were injected with PBS for 30 or 120 min. The results demonstrated that AzA stimulated pCREB by 2-fold after a 2 h injection of AzA (Figures 5A,B). The expression of PGC-1α upon AzA treatment was also upregulated by approximately 1.5-fold in soleus muscle tissues (Figures 5A,C). However, the expressions of pCREB and PGC-1α were unaffected in skeletal muscle tissues of Olfr544-deficient mice (Figures 5B,C). These results demonstrated that AzA might regulate mitochondrial biogenesis in skeletal muscle tissues through activation of the Olfr544-CREB-PGC-1α signaling axis.

Additionally, the levels of pERK and the pERK-to-ERK ratio were also significantly increased by 3.0-fold in soleus muscle tissues after 30 min of AzA treatment (Figures 5A,D). The LC3-II-to-LC3-I protein expression ratio was increased by 1.5-fold after 30 min of AzA; however, these inductions were negated in the skeletal muscle of Olfr544 knockout mice (Figures 5A,E). These suggest that AzA-dependent Olfr544 activation in skeletal muscle tissues induces mitochondrial biogenesis by activation of CREB-PGC-1α and stimulates autophagy formation.

### Oral Administration of AzA Activates Mitochondrial Biogenesis in HFD-Induced Obese Mice

Finally, we investigated metabolic effect of AzA administration on skeletal muscle tissue in HFD-induced obese mice. Obesity is inversely associated with mitochondrial replication and skeletal muscle function, which are caused by cellular oxidative stress, lipotoxicity, and insulin resistance (Holloway et al., 2009; Yan et al., 2012). Skeletal muscle in obese mice and humans increases intramuscular triglyceride concentrations while reducing the rate of lipid oxidation by impairing mitochondrial enzymes (Gerhart-Hines et al., 2007; Holloway et al., 2009).

**FIGURE 6:**
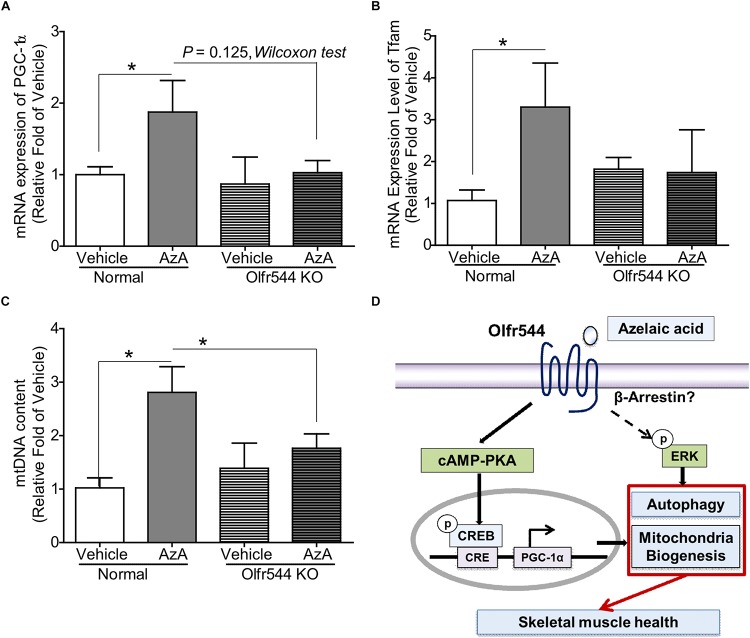
Oral administration of AzA activates mitochondrial biogenesis in skeletal muscle tissues in HFD-induced obese mice. **(A)** AzA induced Pgc-1α gene expression in wild-type mouse skeletal muscle tissues but not in those of Olfr544 KO mice (*n* = 4). **(B,C)** Gene expression of the mitochondrial marker Tfam and mtDNA content were measured by real-time qPCR (*n* = 4). **(D)** Schematic illustration proposing the mechanism by which AzA-driven Olfr544 activation induces mitochondrial biogenesis in skeletal muscle cells by stimulation of CREB-PGC-1α signaling and ERK1/2 activity. Data are the mean ± SEM. Data are statistically significant different denoted by * for *P* ≤ 0.05 using Wilcoxon test and one-way ANOVA followed by Tukey’s HSD test.

Both wild-type and Olfr544 knockout mice were fed HFD to induce obesity and were then orally administered AzA (50 mg/kg body weight/day) for 6 weeks. Body weight and plasma glucose and triglyceride concentrations were reduced and glucose tolerance improved by AzA administration in wild-type mice but not in Olfr544 knockout mice, as reported previously (Wu et al., 2017). The mRNA expression of PGC-1α was induced by 2.0-fold in AzA soleus muscles compared with those in control mice. In contrast, the induction was abrogated in Olfr544 knockout mice (Figure 6A). Herein, we got the significant difference of PGC-1α mRNA expression between AzA-administrating WT mice compared to vehicle mice. We also observed the reduced trend in AzA-administrating Olfr544 KO mice compared to that of WT mice (Figure 6A) although *p* = 0.07. Similarly, expression of the downstream transcriptional target of PGC-1α, mitochondria transcription factor A (*Tfam*), which indicates mitochondrial replication and function, was substantially induced by 3.0-fold in AzA administered wild-type mice, whereas these inductions were negated in skeletal muscles of Olfr544 knockout mice (Figure 6B). The mtDNA content of AzA-administered wild-type skeletal muscle significantly increased by approximately 3.0- and 2.0-fold compared with vehicle-treated wild-type and AzA-administered Olfr544 knockout skeletal muscle, respectively (Figure 6C). In contrast, the mtDNA content was not altered by AzA administration in Olfr544 knockout mice (Figure 6C). Collectively, these data demonstrate that AzA stimulates mitochondrial biogenesis and mitochondrial contents in skeletal muscle tissues via activation of Olfr544 (Figure 6D).

## Discussion

Mitochondrial dysfunction has been suggested to be causally involved in obesity-induced insulin resistance and in the pathophysiology of type II diabetes (T2D). This raised the possibility that mitochondria in skeletal muscle cells could be targets to prevent type 2 diabetes mellitus (Goodpaster, 2013; Hesselink et al., 2016), and these biological processes can be regulated by natural substances and food molecules.

AzA s contained in several grain food (oat, barley, etc.) and can be endogenously synthesized by ω-oxidation process as an end product of linoleic acid. Thus, we believe that Olfr544 in extra-nasal tissues such as skeletal muscle can be endogenously stimulated by AzA derived from diet or endogenous synthesis. In our previous studies, AzA levels were particularly increased in fasting state compared with those in fed state (Wu et al., 2017), thus we suggested that AzA is a redundant fasting signaling molecule that can activate Olfr544 in multiple tissue. Previously we reported that Olfr544 activation by AzA induces white adipose lipolysis, brown adipose thermogenesis, and hepatic fatty acid oxidation (Wu et al., 2017). In this study, we found additional function of Olfr5444, the activation of mitochondrial biogenesis in skeletal muscle. AzA has been detected in humans (Bondia-Pons et al., 2013) and AzA treated human adipose cells showed induced lipolysis, suggesting that AzA has similar functions in humans as well. Biological effects of AzA have been reported. Toxicity studies of AzA have been reported *in vivo*, and the oral LD50 in rats is > 5 g/Kg (Thermo Fisher, MSDS). Pharmacokinetic studies revealed that, in the case of oral administration, approximately 60% of the systemically absorbed AzA is eliminated unchanged through the kidneys. After an intravenous dose, approximately 80% is excreted in the urine within 12 h of administration (Gollnick and Layton, 2008; Sieber and Hegel, 2014). In healthy humans, plasma AzA can reach up to 75 mg/L after 2 h of oral administration of 0.5–5 g (Fitton and Goa, 1991).

Several physiological effects of AzA have been reported. AzA promotes the reduction of lipid peroxides into lipid hydroxides, preventing cardiovascular diseases (Raghavamenon et al., 2009). Animal feeding studies have reported that AzA can reduce atherosclerosis and diabetes phenotypes with the reduction of plasma triglycerides and glucose concentrations and the improvement of glucose tolerance. AzA has been reported to ameliorate glucose metabolism and cholesterol plaque formation in the arteries when administered orally (Muthulakshmi and Saravanan, 2013). AzA administration reduced plasma glucose, insulin, liver glycogen and key carbohydrate metabolic enzymes in HFD-induced type 2 diabetic mice (Litvinov et al., 2010; Muthulakshmi and Saravanan, 2013). These data suggest that AzA may have preventive and therapeutic potential for the treatment of obesity-induced T2DM. We have also reported that activation of Olfr544 by AzA stimulates fatty acid oxidation in hepatocytes and brown adipose tissue, resulting in the reduction of adiposity and the rewiring of fuel preferences toward fats in obese mice (Wu et al., 2017). These findings suggest that Olfr544 can respond to AzA and stimulate cellular energy metabolism under physiological pathways in a variety of tissue types, especially skeletal muscle.

Olfactory receptor signaling pathways and their downstream molecular effectors may serve as effective pharmacologic targets for improving both muscle physiology and the efficiency of cells (Jean-Baptiste et al., 2005; Griffin et al., 2009). In the present study, we demonstrate that Olfr544-dependent PGC-1α and ERK1/2 stimulation is involved in skeletal muscle mitochondria in response to AzA stimulation *in vitro* and by oral administration in skeletal muscle tissues. To the best of our knowledge, this is the first report of ectopic functional expression of ORs on mitochondrial biogenesis in skeletal muscle. Olfr544 activation by AzA induced both mitochondrial biogenesis and autophagy via ERK-LC3II activation. This autophagy can stimulate mitochondrial biogenesis coupled with the removal of damaged and unhealthy mitochondria (Lee et al., 2012).

Mitochondrial biogenesis can be induced by exercise training or exercise mimetics via activation of PGC-1α (Narkar et al., 2008; Qi and Ding, 2012; Wenz, 2013). Activated PGC-1α regulates gene expression, encoding proteins related to mitochondrial biogenesis, oxidative respiration in muscle fibers, and exercise-induced autophagy (Wu et al., 1999; Handschin and Spiegelman, 2011; Lira et al., 2013). Moreover, PGC-1α expression can induce gene expression of an insulin-sensitive glucose transporter that enhances glucose uptake in skeletal muscle cells (Michael et al., 2001). Meanwhile, autophagy is involved in the turnover of mitochondria and other cellular organelles (Wang and Klionsky, 2011). Autophagy results in enhanced oxidative metabolism in muscle and is required for endurance exercise training-induced skeletal muscle adaption by mitochondrial biogenesis induction, which improves physical performance (Lira et al., 2013). Therefore, the enhancement of mitochondrial biogenesis and autophagy in muscle can increase skeletal and brown fat mass that consequently increases energy expenditure and reduces diet-induced obesity.

ERKs regulate both mitochondrial biogenesis and autophagy (Sivaprasad and Basu, 2008; Echave et al., 2009; Cagnol and Chambard, 2010; Wang et al., 2014). The localization of phosphorylated ERK2 to the mitochondria is tightly correlated with autophagic/mitophagic cell stress (Dagda et al., 2008). It has been shown that several GPCR proteins including olfactory receptors stimulate ERK phosphorylation by β-arrestin-dependent manners (Bourquard et al., 2015; Eishingdrelo et al., 2015).

Activation of ERK1/2 subsequently triggers phosphorylation of a number of downstream targets that regulate the autophagy pathway. ERK1/2 phosphorylation has been shown to enhance autophagy in Silymarin-treated Beas-2B cells or mediate phosphorylated Bcl-2 regulated starvation-induced autophagy (Tang et al., 2010; Li et al., 2016). It has been shown that MEK-ERK inhibitors, such as U0126, or amino acids can inhibit autophagy (Pattingre et al., 2003; Tang et al., 2010). Several recent studies have reported that ERK-mTOR signaling may play a major role in autophagy regulation. It has been suggested that transiently or moderately activated ERK1/2 inhibits mTOR activity, which improves cytoprotective autophagy (Wang et al., 2009). Recently, Martinez-Lopez et al. (2013), revealed that ERK1/2 phosphorylation could be used to determine the cellular availability of autophagic structures because LC3 II-positive membranes in pre-autophagosomes might promote coordination of the MEK-ERK1/2 signaling cascade. Here, we observed that AzA-driven Olfr544 activation increased ERK1/2 activity both *in vitro* and *in vivo* followed by the partial induction of LC3-II-to-LC3-I conversion, a marker of autophagy. However, the ratio LC3-II-to-LC3-I were decreased in mice with acute AzA injection for 120 min, indicating alternative pathways may be involved in the regulation of AzA on autophagy. Nonetheless, the detailed mechanism by which AzA-activated Olfr544 induces autophagy in skeletal muscle is required for further studies.

In this study, we investigated acute effect of Olfr544 activation by AzA in both wild-type and Olfr544 KO mice and there was no HFD group (Figure 5). We also administered AzA orally in HFD fed wild-type and Olfr544 KO mice and there was no chow diet group (Figure 6). It should have been better to include both chow and HFD groups in studies of Figures 5, 6, however, our major interest in experiments in Figures 5, 6 were to examine the effect of Olfr544 activation but not to find the effect of HFD. However, the comparison between normal chow and HFD has been investigated by other researchers. It has been reported that HFD affects expression of genes involved in mitochondrial function and biogenesis (Lauren et al., 2005; Cory et al., 2016). ERK levels and the LC3-II/I ratios are induced in HFD-fed skeletal muscle (Cory et al., 2016). Moreover, a 90 and 40% reduction in mRNA and protein levels, respectively, were observed for Pgc1α after 3-week HFD (Lauren et al., 2005).

Skeletal muscle mitochondria are required for muscle physical performance and are beneficial for treating obesity and obesity-induced T2D owing to their lipid oxidation and glycolytic energy capacities (Rogge, 2009; Gouspillou and Hepple, 2016; Hesselink et al., 2016). Our findings demonstrate a novel function of olfactory receptor Olfr544 in skeletal muscle mitochondrial homeostasis. Olfr544 activation contributions to mitochondria biogenesis via PKA-CREB-PGC-1α and ERK-LC3II signaling (Figure 6D) in skeletal muscle. These data also suggest that Olfr544 may be a potential target to stimulate skeletal muscle function.

## Data Availability Statement

All datasets generated for this study are included in the article/Supplementary Material.

## Ethics Statement

The animal study was reviewed and approved by the Animal Experiment Committee of Korea University (Protocol No. KUIACUC-2019-0031).

## Author Contributions

S-JL designed the research. TT and CW performed the experiments and analyzed the data. S-JL and KH discussed the interpretation of the results. TT, CW, and S-JL wrote the manuscript.

## Conflict of Interest

The authors declare that the research was conducted in the absence of any commercial or financial relationships that could be construed as a potential conflict of interest.

## Abbreviations

Azelaic acid: AzA
cyclic adenosine monophosphate: cAMP
cAMP-response element binding protein: CREB
extracellular signal-regulated kinase-1/2: ERK1/2
G-protein coupled receptors: GPCR
high fat diet: HFD
mitochondrial transcription factor A: TFAM
olfactory receptors: ORs
olfactory receptor 544: Olfr544
protein kinase A: PKA
peroxisome proliferator-activated receptor gamma coactivator 1-alpha: PGC-1 α
small interfering RNA: siRNA.

## References

[B1] Bondia-Pons I. Barri T. Hanhineva K. Juntunen K. Dragsted L. O. Mykkänen H. (2013). UPLC-QTOF/MS metabolic profiling unveils urinary changes in humans after a whole grain rye versus refined wheat bread intervention. *Mol. Nutr. Food Res.* 57 412–422. 10.1002/mnfr.201200571 23307617

[B2] Bourquard T. Landomiel F. Reiter E. Crepieux P. Ritchie D. W. Aze J. (2015). Unraveling the molecular architecture of a G protein-coupled receptor/beta-arrestin/Erk module complex. *Sci. Rep.* 5:10760. 10.1038/srep10760 26030356 PMC4649906

[B3] Buck L. Axel R. (1991). A novel multigene family may encode odorant receptors: a molecular basis for odor recognition. *Cell* 65 175–187. 1840504 10.1016/0092-8674(91)90418-x

[B4] Cagnol S. Chambard J. C. (2010). ERK and cell death: mechanisms of ERK-induced cell death–apoptosis, autophagy and senescence. *FEBS J.* 277 2–21. 10.1111/j.1742-4658.2009.07366.x 19843174

[B5] Cory M. D. Ji L. David L. W. (2016). Caloric restriction normalizes obesity-induced alterations on regulators of skeletal muscle growth signaling. *Lipid* 51 905–912. 10.1007/s11745-016-4168-3 27289530 PMC5218829

[B6] Dagda R. K. Zhu J. Kulich S. M. Chu C. T. (2008). Mitochondrially localized ERK2 regulates mitophagy and autophagic cell stress: implications for Parkinson’s disease. *Autophagy* 4 770–782. 18594198 10.4161/auto.6458 PMC2574804

[B7] Duclos M. Oppert J. M. Verges B. Coliche V. Gautier J. F. Guezennec Y. (2013). Physical activity and type 2 diabetes. Recommandations of the SFD (Francophone Diabetes Society) diabetes and physical activity working group. *Diabetes Metab.* 39 205–216. 10.1016/j.diabet.2013.03.005 23643351

[B8] Echave P. Machado-da-Silva G. Arkell R. S. Duchen M. R. Jacobson J. Mitter R. (2009). Extracellular growth factors and mitogens cooperate to drive mitochondrial biogenesis. *J. Cell Sci.* 122(Pt 24), 4516–4525. 10.1242/jcs.049734 19920079 PMC2798125

[B9] Eishingdrelo H. Sun W. Li H. Wang L. Eishingdrelo A. Dai S. (2015). ERK and beta-arrestin interaction: a converging point of signaling pathways for multiple types of cell surface receptors. *J. Biomol. Screen.* 20 341–349. 10.1177/1087057114557233 25361946 PMC4975872

[B10] Firestein S. (2001). How the olfactory system makes sense of scents. *Nature* 413 211–218. 10.1038/35093026 11557990

[B11] Fitton A. Goa K. L. (1991). Azelaic acid. A review of its pharmacological properties and therapeutic efficacy in acne and hyperpigmentary skin disorders. *Drugs* 41 780–798. 1712709 10.2165/00003495-199141050-00007

[B12] Gallagher R. S. Ananth R. Granger K. Bradley B. Anderson J. V. Fuerst E. P. (2010). Phenolic and short-chained aliphatic organic acid constituents of wild oat (*Avena fatua* L.) seeds. *J. Agric. Food Chem.* 58 218–225. 10.1021/jf9038106 20017486

[B13] Gerhart-Hines Z. Rodgers J. T. Bare O. Lerin C. Kim S. H. Mostoslavsky R. (2007). Metabolic control of muscle mitochondrial function and fatty acid oxidation through SIRT1/PGC-1alpha. *EMBO J.* 26 1913–1923. 10.1038/sj.emboj.7601633 17347648 PMC1847661

[B14] Gollnick H. Layton A. (2008). Azelaic acid 15% gel in the treatment of rosacea. *Expert Opin. Pharmacother.* 9 2699–2706. 10.1517/14656566.9.15.2699 18803456

[B15] Goodpaster B. H. (2013). Mitochondrial deficiency is associated with insulin resistance. *Diabetes Metab. Res. Rev.* 62 1032–1035. 10.2337/db12-1612 PMC3609595 23520282

[B16] Gouspillou G. Hepple R. T. (2016). Editorial: mitochondria in skeletal muscle health, aging and diseases. *Front. Physiol.* 7:446 10.3389/fphys.2016.00446 PMC5052271 27766080

[B17] Griffin C. A. Kafadar K. A. Pavlath G. K. (2009). MOR23 promotes muscle regeneration and regulates cell adhesion and migration. *Dev. Cell* 17 649–661. 10.1016/j.devcel.2009.09.004 19922870 PMC2780437

[B18] Handschin C. Spiegelman B. M. (2011). PGC-1 coactivators and the regulation of skeletal muscle fiber-type determination. *Cell Metab.* 13:351 10.1016/j.cmet.2011.03.008 21459315

[B19] He C. Bassik M. C. Moresi V. Sun K. Wei Y. Zou Z. (2012). Exercise-induced BCL2-regulated autophagy is required for muscle glucose homeostasis. *Nature* 481 511–515. 10.1038/nature10758 22258505 PMC3518436

[B20] Herzig S. Long F. Jhala U. S. Hedrick S. Quinn R. Bauer A. (2001). CREB regulates hepatic gluconeogenesis through the coactivator PGC-1. *Nature* 413 179–183. 10.1038/35093131 11557984

[B21] Hesselink M. K. Schrauwen-Hinderling V. Schrauwen P. (2016). Skeletal muscle mitochondria as a target to prevent or treat type 2 diabetes mellitus. *Nat. Rev. Endocrinol.* 12 633–645. 10.1038/nrendo.2016.104 27448057

[B22] Hoang M. H. Jia Y. Mok B. Jun H. J. Hwang K. Y. Lee S. J. (2015). Kaempferol ameliorates symptoms of metabolic syndrome by regulating activities of liver X receptor-beta. *J. Nutr. Biochem.* 26 868–875. 10.1016/j.jnutbio.2015.03.005 25959373

[B23] Holloway G. P. Bonen A. Spriet L. L. (2009). Regulation of skeletal muscle mitochondrial fatty acid metabolism in lean and obese individuals. *Am. J. Clin. Nutr.* 89 455S–462S. 10.3945/ajcn.2008.26717B 19056573

[B24] Jean-Baptiste G. Yang Z. Khoury C. Gaudio S. Greenwood M. T. (2005). Peptide and non-peptide G-protein coupled receptors (GPCRs) in skeletal muscle. *Peptides* 26 1528–1536. 10.1016/j.peptides.2005.03.011 16042993

[B25] Jia Y. Kim J. Y. Jun H. J. Kim S. J. Lee J. H. Hoang M. H. (2013). Cyanidin is an agonistic ligand for peroxisome proliferator-activated receptor-alpha reducing hepatic lipid. *Biochim. Biophys. Acta* 1831 698–708. 10.1016/j.bbalip.2012.11.012 23228689

[B26] Jia Y. Kim S. Kim J. Kim B. Wu C. Lee J. H. (2015). Ursolic acid improves lipid and glucose metabolism in high-fat-fed C57BL/6J mice by activating peroxisome proliferator-activated receptor alpha and hepatic autophagy. *Mol. Nutr. Food Res.* 59 344–354. 10.1002/mnfr.201400399 25418615

[B27] Jia Y. Wu C. Kim J. Kim B. Lee S. J. (2016). Astaxanthin reduces hepatic lipid accumulations in high-fat-fed C57BL/6J mice via activation of peroxisome proliferator-activated receptor (PPAR) alpha and inhibition of PPAR gamma and Akt. *J. Nutr. Biochem.* 28 9–18. 10.1016/j.jnutbio.2015.09.015 26878778

[B28] Jun H. J. Lee J. H. Kim J. Jia Y. Kim K. H. Hwang K. Y. (2014). Linalool is a PPAR alpha ligand that reduces plasma TG levels and rewires the hepatic transcriptome and plasma metabolome. *J. Lipid Res.* 55 1098–1110. 10.1194/jlr.M045807 24752549 PMC4031941

[B29] Kang N. Bahk Y. Y. Lee N. Jae Y. Cho Y. H. Ku C. R. (2015). Olfactory receptor Olfr544 responding to azelaic acid regulates glucagon secretion in alpha-cells of mouse pancreatic islets. *Biochem. Biophys. Res. Commun.* 460 616–621. 10.1016/j.bbrc.2015.03.078 25804639

[B30] Lauren M. S. Hui X. Robert A. K. Randall M. Matthew W. H. George A. B. (2005). A high-fat diet coordinately downregulates genes required for mitochondrial oxidative phosphorylation in skeletal muscle. *Diabetes Metab. Res. Rev.* 54 1926–1933. 15983191 10.2337/diabetes.54.7.1926

[B31] Lee J. Giordano S. Zhang J. (2012). Autophagy, mitochondria and oxidative stress: cross-talk and redox signalling. *Biochem. J.* 441 523–540. 10.1042/BJ20111451 22187934 PMC3258656

[B32] Lee S. J. Depoortere I. Hatt H. (2019). Therapeutic potential of ectopic olfactory and taste receptors. *Nat. Rev. Drug Discov.* 18 116–138. 10.1038/s41573-018-0002-3 30504792

[B33] Li D. Hu J. Wang T. Zhang X. Liu L. Wang H. (2016). Silymarin attenuates cigarette smoke extract-induced inflammation via simultaneous inhibition of autophagy and ERK/p38 MAPK pathway in human bronchial epithelial cells. *Sci. Rep.* 6:37751. 10.1038/srep37751 27874084 PMC5118787

[B34] Lira V. A. Okutsu M. Zhang M. Greene N. P. Laker R. C. Breen D. S. (2013). Autophagy is required for exercise training-induced skeletal muscle adaptation and improvement of physical performance. *FASEB J.* 27 4184–4193. 10.1096/fj.13-228486 23825228 PMC4046188

[B35] Little J. P. Cochran A. J. (2011). Regulating the regulators: the role of transcriptional regulatory proteins in the adaptive response to exercise in human skeletal muscle. *J. Physiol.* 589(Pt 7), 1511–1512. 10.1113/jphysiol.2011.205401 21486836 PMC3099009

[B36] Litvinov D. Selvarajan K. Garelnabi M. Brophy L. Parthasarathy S. (2010). Anti-atherosclerotic actions of azelaic acid, an end product of linoleic acid peroxidation, in mice. *Atherosclerosis* 209 449–454. 10.1016/j.atherosclerosis.2009.09.076 19880116 PMC2846213

[B37] Lo Verso F. Carnio S. Vainshtein A. Sandri M. (2014). Autophagy is not required to sustain exercise and PRKAA1/AMPK activity but is important to prevent mitochondrial damage during physical activity. *Autophagy* 10 1883–1894. 10.4161/auto.32154 25483961 PMC4502666

[B38] Martinez-Lopez N. Athonvarangkul D. Mishall P. Sahu S. Singh R. (2013). Autophagy proteins regulate ERK phosphorylation. *Nat. Commun.* 4:2799. 10.1038/ncomms3799 24240988 PMC3868163

[B39] McConell G. K. Ng G. P. Phillips M. Ruan Z. Macaulay S. L. Wadley G. D. (2010). Central role of nitric oxide synthase in AICAR and caffeine-induced mitochondrial biogenesis in L6 myocytes. *J. Appl. Physiol.* 108 589–595. 10.1152/japplphysiol.00377.2009 20044477

[B40] Michael L. F. Wu Z. Cheatham R. B. Puigserver P. Adelmant G. Lehman J. J. (2001). Restoration of insulin-sensitive glucose transporter (GLUT4) gene expression in muscle cells by the transcriptional coactivator PGC-1. *Proc. Natl. Acad. Sci. U.S.A.* 98 3820–3825. 10.1073/pnas.061035098 11274399 PMC31136

[B41] Muthulakshmi S. Saravanan R. (2013). Efficacy of azelaic acid on hepatic key enzymes of carbohydrate metabolism in high fat diet induced type 2 diabetic mice. *Biochimie* 95 1239–1244. 10.1016/j.biochi.2013.01.018 23402910

[B42] Narkar V. A. Downes M. Yu R. T. Embler E. Wang Y. X. Banayo E. (2008). AMPK and PPARdelta agonists are exercise mimetics. *Cell* 134 405–415. 10.1016/j.cell.2008.06.051 18674809 PMC2706130

[B43] Pattingre S. Bauvy C. Codogno P. (2003). Amino acids interfere with the ERK1/2-dependent control of macroautophagy by controlling the activation of Raf-1 in human colon cancer HT-29 cells. *J. Biol. Chem.* 278 16667–16674. 10.1074/jbc.M210998200 12609989

[B44] Perry C. G. R. Hawley J. A. (2018). Molecular basis of exercise-induced skeletal muscle mitochondrial biogenesis: historical advances. current knowledge, and future challenges. *Cold Spring Harb. Perspect. Med.* 8:a029686. 10.1101/cshperspect.a029686 28507194 PMC6120690

[B45] Qi Z. Ding S. (2012). Transcriptional regulation by nuclear corepressors and PGC-1alpha: implications for mitochondrial quality control and insulin sensitivity. *PPAR Res.* 2012:348245. 10.1155/2012/348245 23304112 PMC3523614

[B46] Raghavamenon A. Garelnabi M. Babu S. Aldrich A. Litvinov D. Parthasarathy S. (2009). Alpha-tocopherol is ineffective in preventing the decomposition of preformed lipid peroxides and may promote the accumulation of toxic aldehydes: a potential explanation for the failure of antioxidants to affect human atherosclerosis. *Antioxid. Redox. Signal.* 11 1237–1248. 10.1089/ARS.2008.2248 19186999 PMC2842134

[B47] Rogge M. M. (2009). The role of impaired mitochondrial lipid oxidation in obesity. *Biol. Res. Nurs.* 10 356–373. 10.1177/1099800408329408 19190032

[B48] Russell A. P. Foletta V. C. Snow R. J. Wadley G. D. (2014). Skeletal muscle mitochondria: a major player in exercise, health and disease. *Biochim. Biophys. Acta* 1840 1276–1284. 10.1016/j.bbagen.2013.11.016 24291686

[B49] Scarpulla R. C. Vega R. B. Kelly D. P. (2012). Transcriptional integration of mitochondrial biogenesis. *Trends Endocrinol. Metab.* 23 459–466. 10.1016/j.tem.2012.06.006 22817841 PMC3580164

[B50] Schmidt S. F. Mandrup S. (2011). Gene program-specific regulation of PGC-1{alpha} activity. *Genes Dev.* 25 1453–1458. 10.1101/gad.2076411 21764849 PMC3143934

[B51] Sieber M. A. Hegel J. K. (2014). Azelaic acid: properties and mode of action. *Skin Pharmacol. Physiol.* 27(Suppl. 1), 9–17. 10.1159/000354888 24280644

[B52] Sivaprasad U. Basu A. (2008). Inhibition of ERK attenuates autophagy and potentiates tumour necrosis factor-alpha-induced cell death in MCF-7 cells. *J. Cell Mol. Med.* 12 1265–1271. 10.1111/j.1582-4934.2008.00282.x 18266953 PMC3865671

[B53] Tang D. Kang R. Livesey K. M. Cheh C. W. Farkas A. Loughran P. (2010). Endogenous HMGB1 regulates autophagy. *J. Cell Biol.* 190 881–892. 10.1083/jcb.200911078 20819940 PMC2935581

[B54] Thach T. T. Lee C. K. Park H. W. Lee S. J. Lee S. J. (2016). Syringaresinol induces mitochondrial biogenesis through activation of PPARbeta pathway in skeletal muscle cells. *Bioorg. Med. Chem. Lett.* 26 3978–3983. 10.1016/j.bmcl.2016.07.001 27450788

[B55] Wang J. Whiteman M. W. Lian H. Wang G. Singh A. Huang D. (2009). A non-canonical MEK/ERK signaling pathway regulates autophagy via regulating Beclin 1. *J. Biol. Chem.* 284 21412–21424. 10.1074/jbc.M109.026013 19520853 PMC2755866

[B56] Wang K. Klionsky D. J. (2011). Mitochondria removal by autophagy. *Autophagy* 7 297–300. 21252623 10.4161/auto.7.3.14502 PMC3359476

[B57] Wang K. Z. Zhu J. Dagda R. K. Uechi G. Cherra S. J. III Gusdon A. M. (2014). ERK-mediated phosphorylation of TFAM downregulates mitochondrial transcription: implications for Parkinson’s disease. *Mitochondrion* 17 132–140. 10.1016/j.mito.2014.04.008 24768991 PMC4134365

[B58] Wenz T. (2013). Regulation of mitochondrial biogenesis and PGC-1alpha under cellular stress. *Mitochondrion* 13 134–142. 10.1016/j.mito.2013.01.006 23347985

[B59] Wright D. C. Geiger P. C. Han D. H. Jones T. E. Holloszy J. O. (2007). Calcium induces increases in peroxisome proliferator-activated receptor gamma coactivator-1alpha and mitochondrial biogenesis by a pathway leading to p38 mitogen-activated protein kinase activation. *J. Biol. Chem.* 282 18793–18799. 10.1074/jbc.M611252200 17488713

[B60] Wu C. Hwang S. H. Jia Y. Choi J. Kim Y. J. Choi D. (2017). Olfactory receptor 544 reduces adiposity by steering fuel preference toward fats. *J. Clin. Invest.* 127 4118–4123. 10.1172/JCI89344 28990936 PMC5663348

[B61] Wu C. Jia Y. Lee J. H. Kim Y. Sekharan S. Batista V. S. (2015). Activation of OR1A1 suppresses PPAR-gamma expression by inducing HES-1 in cultured hepatocytes. *Int. J. Biochem. Cell Biol.* 64 75–80. 10.1016/j.biocel.2015.03.008 25817041

[B62] Wu C. Thach T. T. Kim Y. J. Lee S. J. (2019). Olfactory receptor 43 reduces hepatic lipid accumulation and adiposity in mice. *Biochim. Biophys. Acta Mol. Cell Biol. Lipids* 1864 489–499. 10.1016/j.bbalip.2019.01.004 30639733

[B63] Wu Z. Puigserver P. Andersson U. Zhang C. Adelmant G. Mootha V. (1999). Mechanisms controlling mitochondrial biogenesis and respiration through the thermogenic coactivator PGC-1. *Cell* 98 115–124. 10.1016/S0092-8674(00)80611-X 10412986

[B64] Yan Z. Lira V. A. Greene N. P. (2012). Exercise training-induced regulation of mitochondrial quality. *Exerc. Sport Sci. Rev.* 40 159–164. 10.1097/JES.0b013e3182575599 22732425 PMC3384482

