# Supplementary material for: Azelaic Acid Induces Mitochondrial Biogenesis in Skeletal Muscle by Activation of Olfactory Receptor 544
Source: Front Physiol. 2020 Apr 17;11:329. doi: 10.3389/fphys.2020.00329 (PMC7199515; doi:10.3389/fphys.2020.00329)
Supplement: Supplementary file 1 [file Table_1.DOCX]

#### Supplemental Information

#### Supplemental Methods

**Plasmid and cloning**

DNA encoding the full-length Olfr544 and Olfr545 sequences was amplified from templates (MG215211 and MG213236 plasmids from Origen, respectively) using specific sets of primers (Table S1). The PCR products were isolated from agarose gels and sub-cloned into pMV18 plasmids (kind gift from Dr. Jeniffer Pluznick, Johns Hopkins University) using *EcoRI* and *XhoI* restriction enzymes. The final constructs contain the N-terminal lucy-flag-rho tag. The identities of sequences were confirmed by DNA sequencing. Plasmid was amplified and purified in transformed *E. coli* DH5α cells using a Miniprep kit (Qiagen, Korea).

####

#### *cAMP response element (CRE)-luciferase reporter assay*

Hana3A cells were seeded into 24-well plates to 70-80% confluence. Olfr544 or Olfr545, CRE-firefly reporter and Renilla expression vectors were transiently co-transfected into the cells using Lipofectamine 2000 (Invitrogen, CA, USA) according to the manufacturer’s protocol. Eighteen hours post-transfection, the cells were treated with linear concentrations of AzA for 10 h. A luciferase assay was conducted using a Dual-Glo luciferase assay kit (Promega, WI, USA) and quantified by Victor X2 (PerkinElmer, USA). The CRE-luciferase activity is the normalization of firefly luminescence signal to that of Renilla.

#### Microarray

#### C57BL/6J mice were fed on normal or 60% high-fat diet for 4 weeks prior to isolate tissues. Total RNA from sleketal mucle tissues (soleus muscle) were isolated using RNAiso Plus reagent (Takara Bio, Otsu, Japan). The RNA quality was evaluated using a NanoDrop spectrophotometer (Thermo Scientific, USA) and Experion Automated Electrophoresis Station (Bio-Rad, PA, USA) using Experion RNA Stdsens chips (Bio-Rad, PA, USA). From the NanoDrop results, concentration, 260/280 ratio of the RNA samples were determined and the electropherograms of the Experion data were compared visually with the control samples assessing the ratio of 28S:18S and RNA integrity number. Samples were labeled with Cy3 (ND) and Cy5 (HFD) during reverse transcription and hybridized on a Agilent Mouse GE (V2) 4 X 44K microarray (39,473 probes). Data were normalized with LOWESS method and corrected with backgroud to select 24,040 significantly expressed genes. Microarray hybridization and data analysis were performed by Genomic Works (Daejeon, Korea). The expression of olfactory receptors was analyzed in the dataset. Expression values were summarized after background correction and normalization steps.

**Supplemental Table**

**Table S1. Primer sequences**

| Name | Sequence (5’ → 3’) | Amplicon |  |
| --- | --- | --- | --- |
| **Cloning** |  |  |  |
| Olfr544_EcoRI | ggaattcatgtcagggtggagcaatgg |  | 1181 |
| Olfr544_XhoI | gctcgagtcatccaggaagctctctattcg |  |  |
| Olfr545_EcoRI | ggaattcatgttgggttggagcaatgg |  | 951 |
| Olfr545_XhoI | gctcgagtcaggggcctgcactcagg |  |  |
| **qPCR** |  |  |  |
| Olfr544_fwd | gatgttctgcatctacttccttattg |  | 322 |
| Olfr544_rev | tcggttgaagatgcgaacag |  |  |
| Olfr545_fwd | gatgttctgcatctactttctgg |  | 337 |
| Olfr545_rev | gagcatatccaggactctattaaagg |  |  |
| mtDNA_fwd | cacccagctactaccatcattcaag |  | 118 |
| mtDNA_rev | gatggtttgggagattggttgatg |  |  |
| PGC_1α_fwd | acccacaggatcagaacaaaccc |  | 154 |
| PGC_1α_rev | ttggtgtgaggagggtcatgg |  |  |
| TFAM_fwd | tgggcttagagaaggaagcc |  | 107 |
| TFAM_rev | tgctgaccgaggtctttttg |  |  |
| L32_fwd | ggcctctggtgaagcccaagatcg |  | 107 |
| L32_rev | cctctgggtttccgccagtttcgc |  |  |

**Supplemental Figures**

**
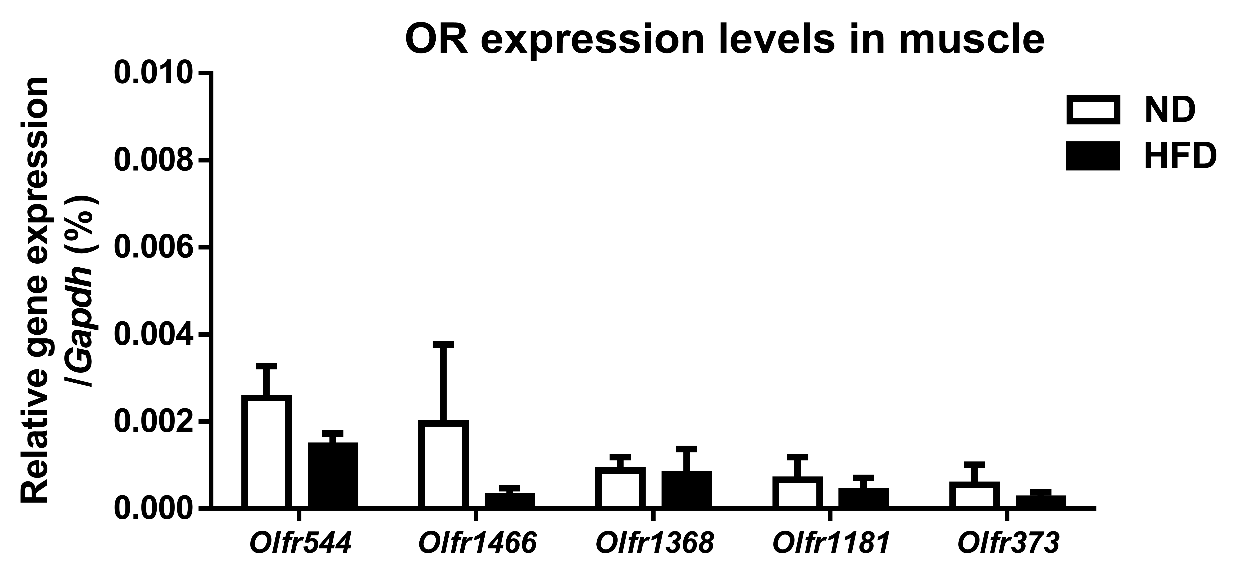
**

**Fig. S1.** Olfactory receptors (ORs) are expressed in mouse muscle tissue. Expression of ORs in mouse muscle tissue assessed by qPCR (n=3–4). The expression of ORs was normalized to that of *Gapdh*. C57BL/6J mice were fed a normal (ND) or a high-fat diet (HFD) for 4 weeks prior to tissue sampling and cDNA preparation. ORs were selected from microarray experiments (data not shown). OR, olfactory receptor; ND, normal diet; HFD, high-fat diet (60% of total calories from fat). Data are presented as mean ± SEM.

**
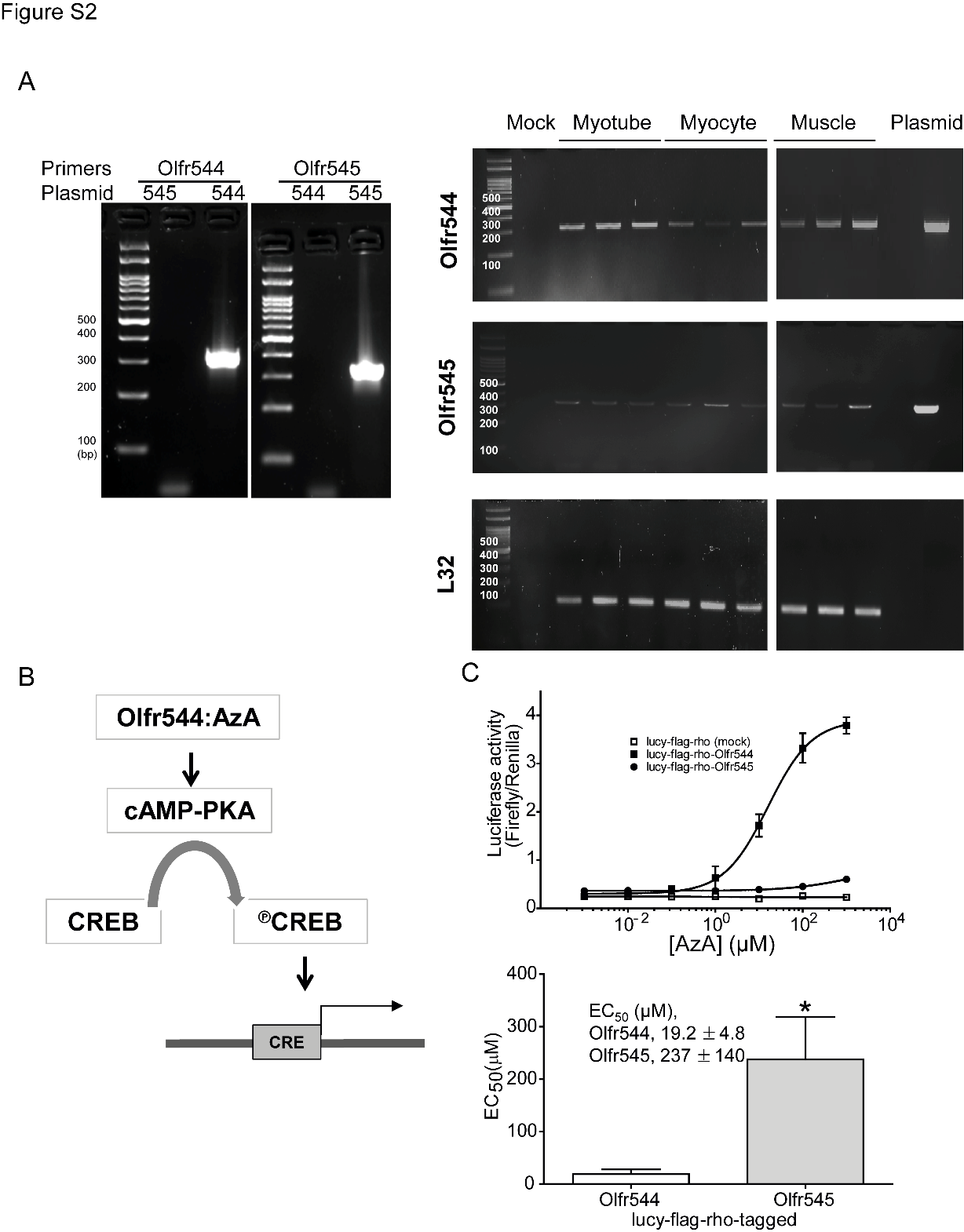
**

**Fig. S2.** Functional expression of Olfr544 in skeletal muscle cells and tissues. (A) Expression of Olfr544 and Olfr545 by RT-PCR. (B) Schematic diagram of CRE-luciferase activity. Hana3A cells treacfected with Olfr544 was stimulated by azelaic acid (AzA) to induce CRE-firefly luciferase reporter gene. (C) Activation of Olfr544 and Olfr545 by AzA and half maximal effective concentration of AzA for Olfr544 and Olfr545. * denotes *P*<0.05.


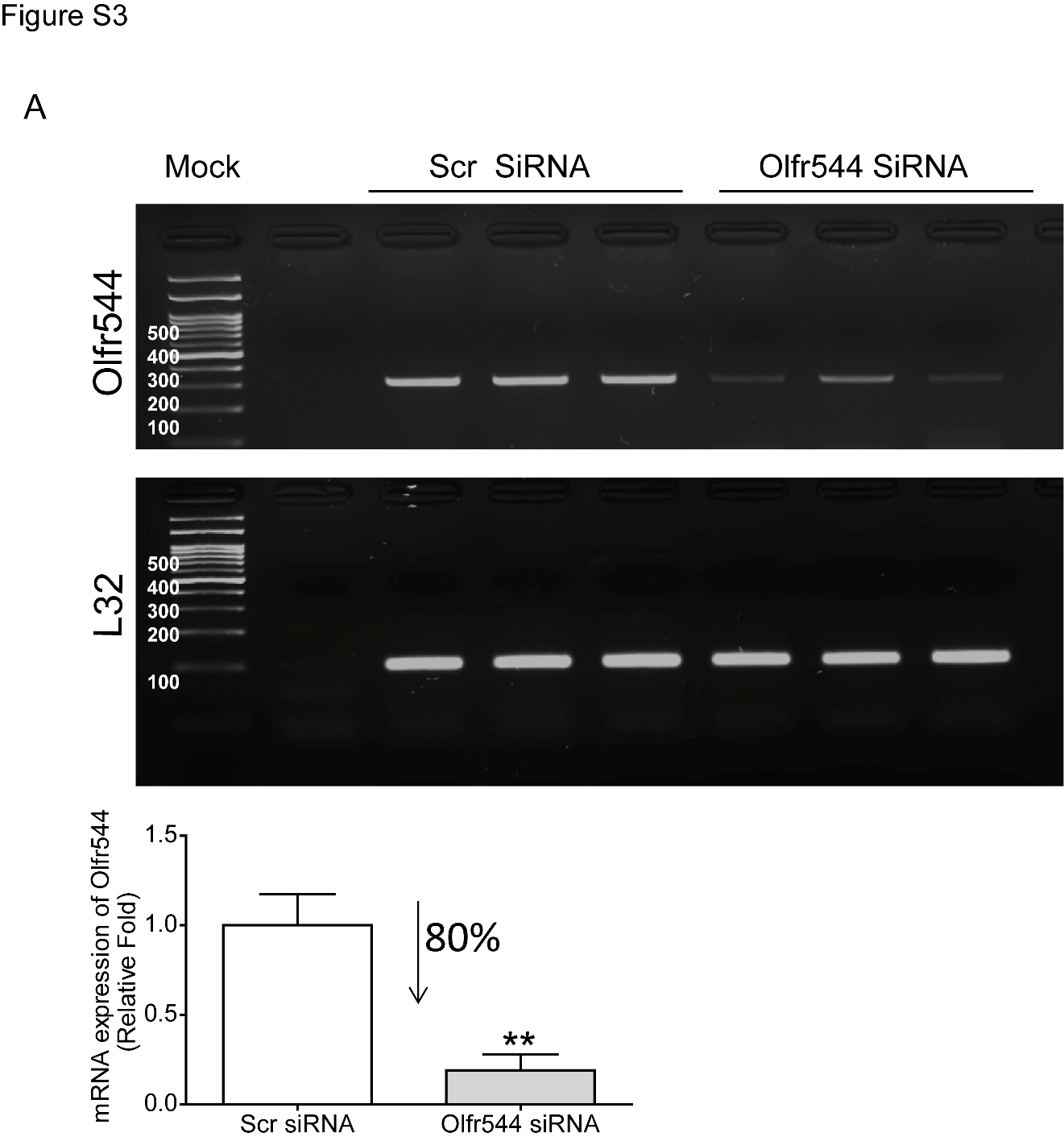


**Fig. S3.** siRNA specific for Olfr544 reduces Olfr544 mRNA expression assessed by RT-PCR. Agarose gel electrophoresis images (top) and normalized expression of Olfr544 (bottom).
