# Supplementary material for: Azelaic Acid Induces Mitochondrial Biogenesis in Skeletal Muscle by Activation of Olfactory Receptor 544
Source: Front Physiol. 2020 Apr 17;11:329. doi: 10.3389/fphys.2020.00329 (PMC7199515; doi:10.3389/fphys.2020.00329)
Supplement: Supplementary file 2 [file Presentation_1.PPTX]

## Slide 1
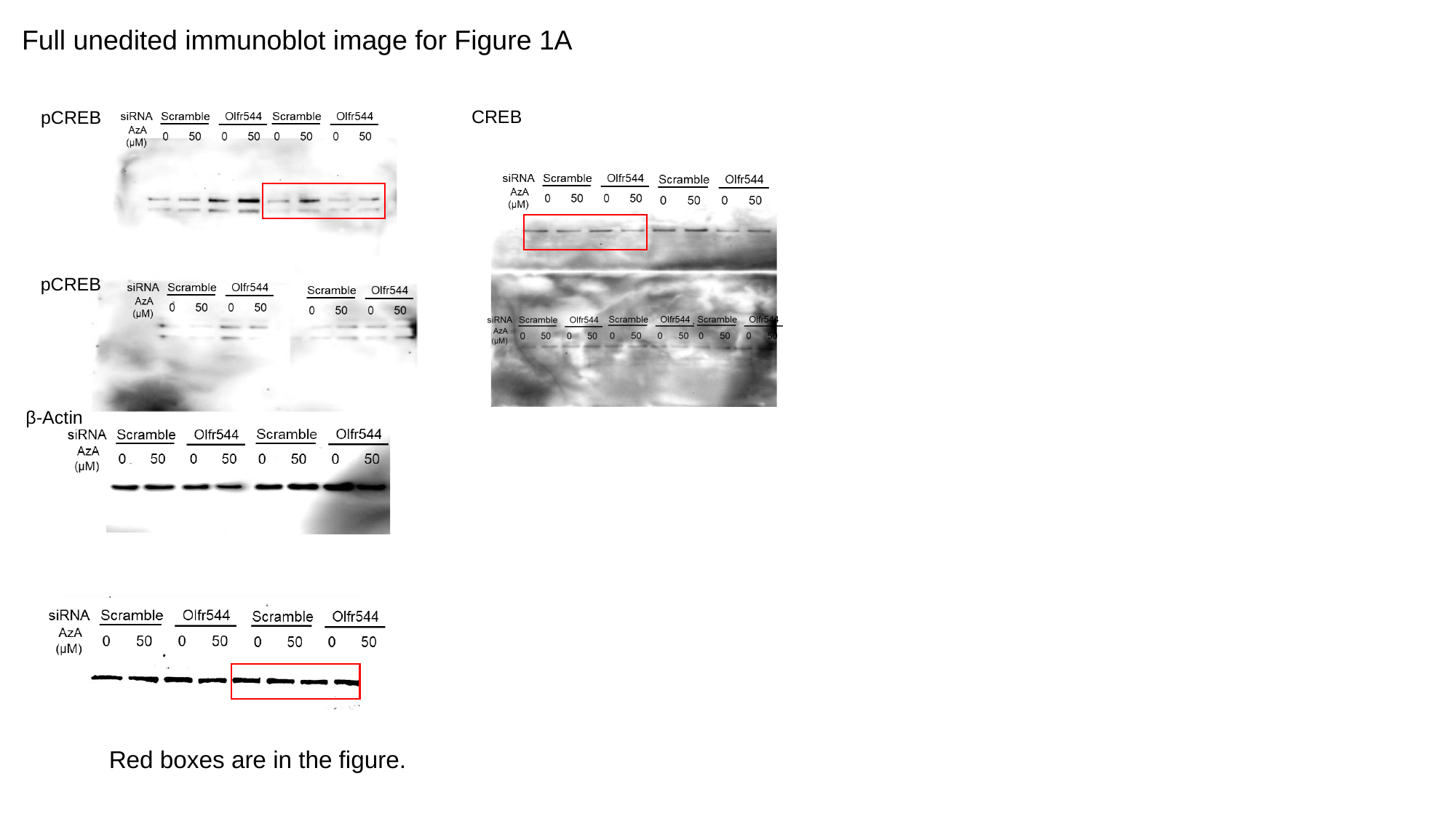

Full unedited immunoblot image for Figure 1A
CREB
pCREB
pCREB
pCREB
β-Actin
Red boxes are in the figure.

## Slide 2
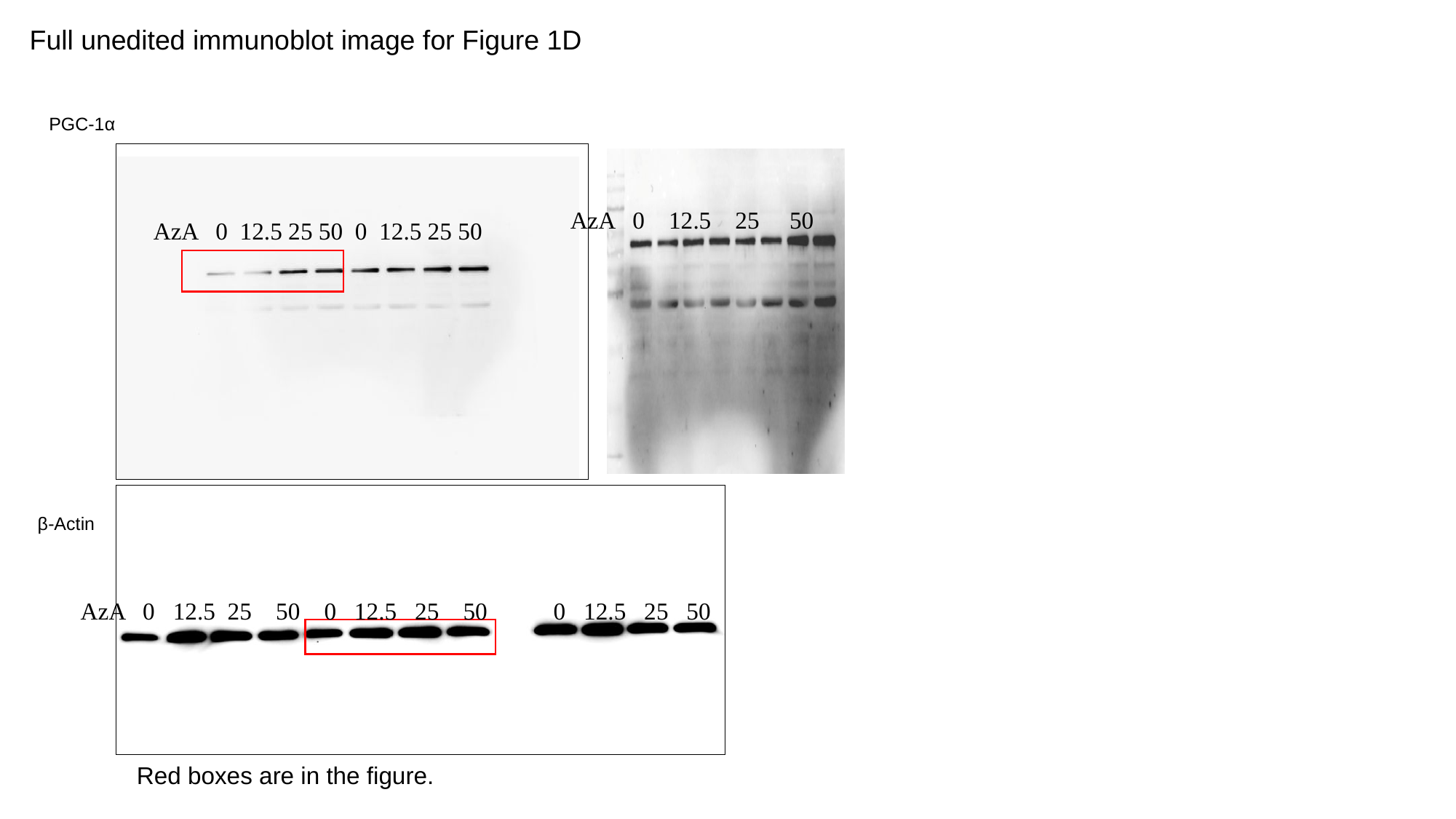

Full unedited immunoblot image for Figure 1D
PGC-1α
AzA 0 12.5 25 50
AzA 0 12.5 25 50 0 12.5 25 50
β-Actin
AzA 0 12.5 25 50 0 12.5 25 50 0 12.5 25 50
Red boxes are in the figure.

## Slide 3
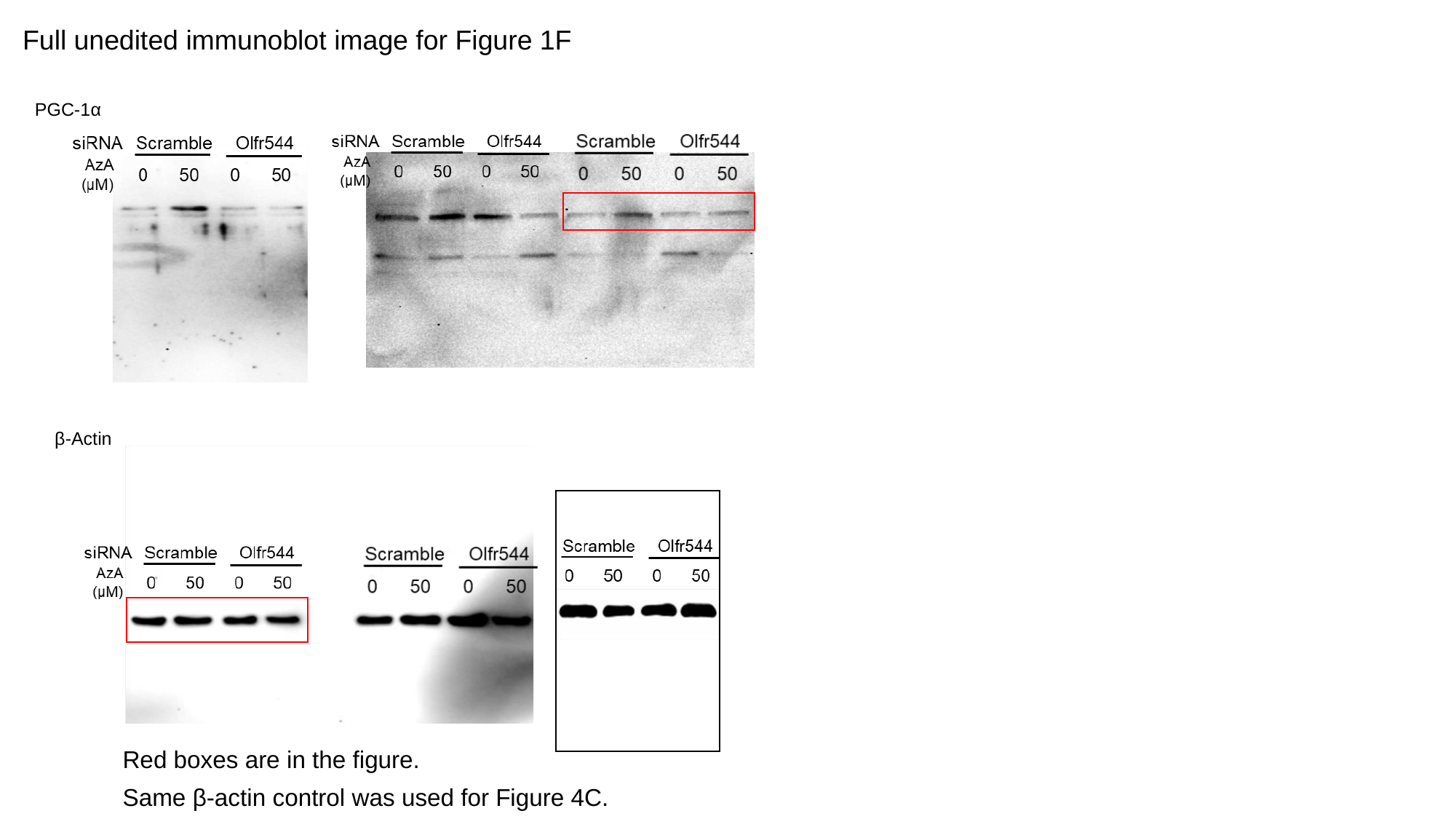

Full unedited immunoblot image for Figure 1F
PGC-1α
β-Actin
Red boxes are in the figure.
Same β-actin control was used for Figure 4C.

## Slide 4
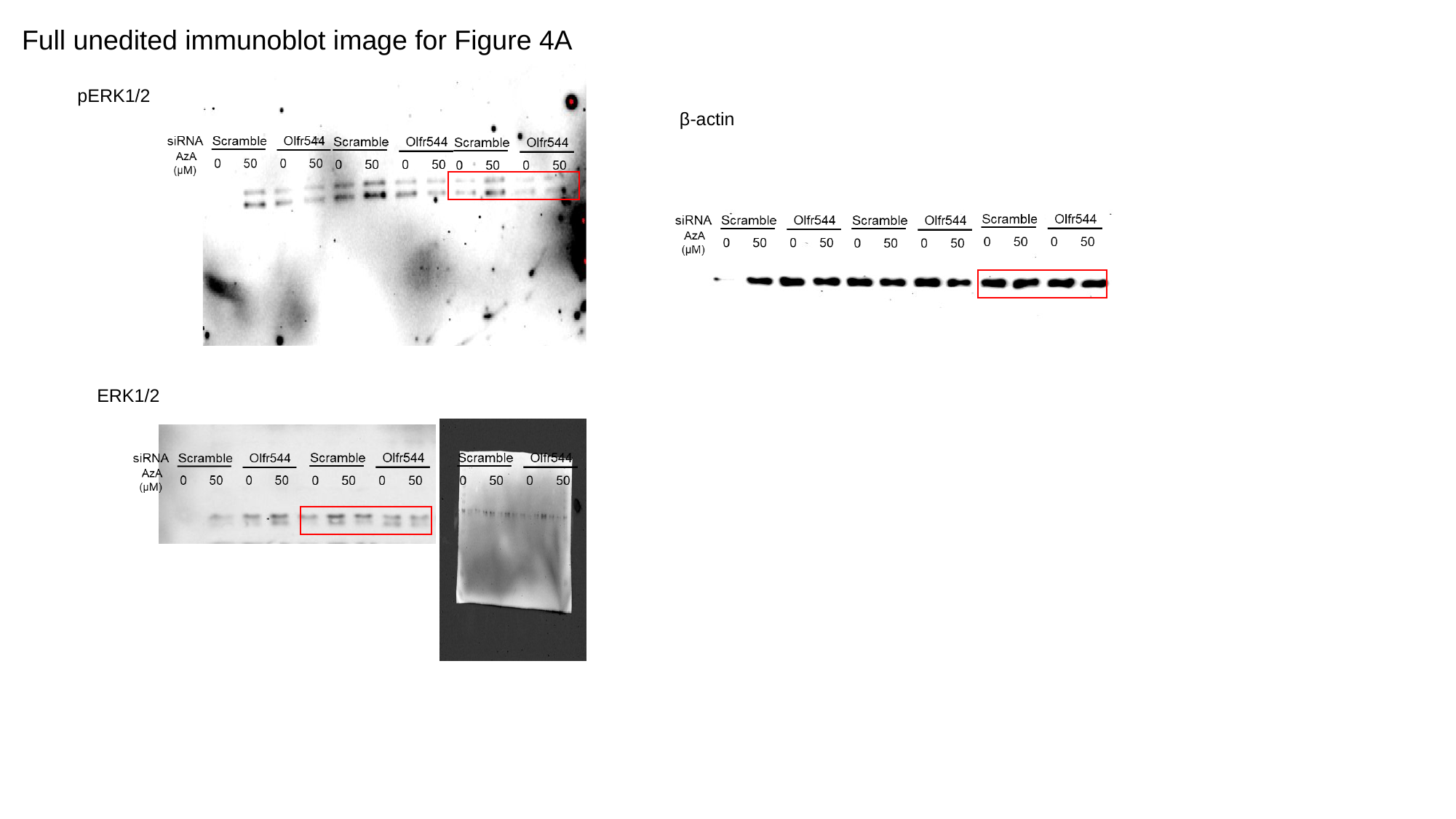

Full unedited immunoblot image for Figure 4A
pERK1/2
β-actin
ERK1/2

## Slide 5
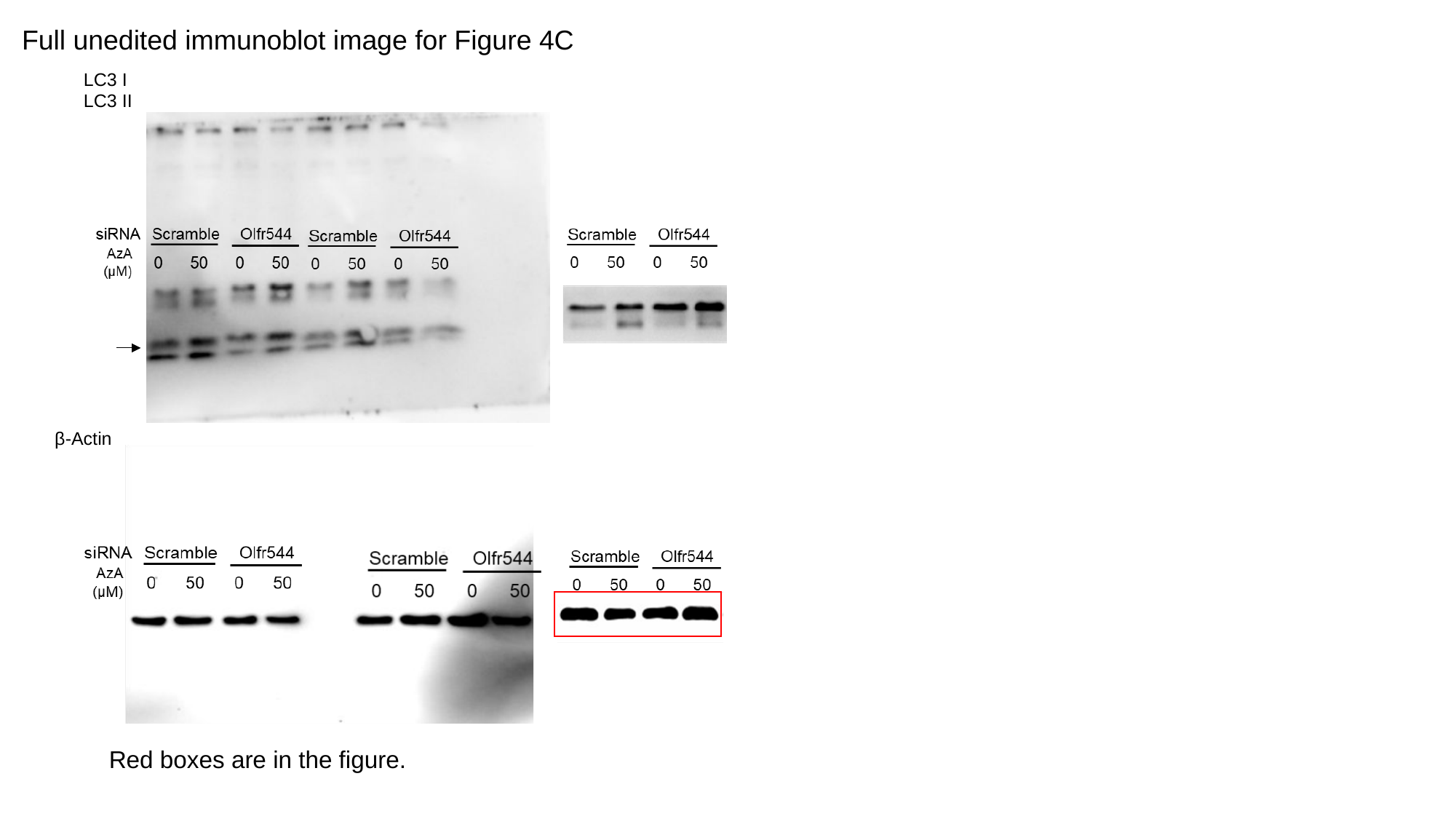

Full unedited immunoblot image for Figure 4C
LC3 I
LC3 II
β-Actin
Red boxes are in the figure.

## Slide 6
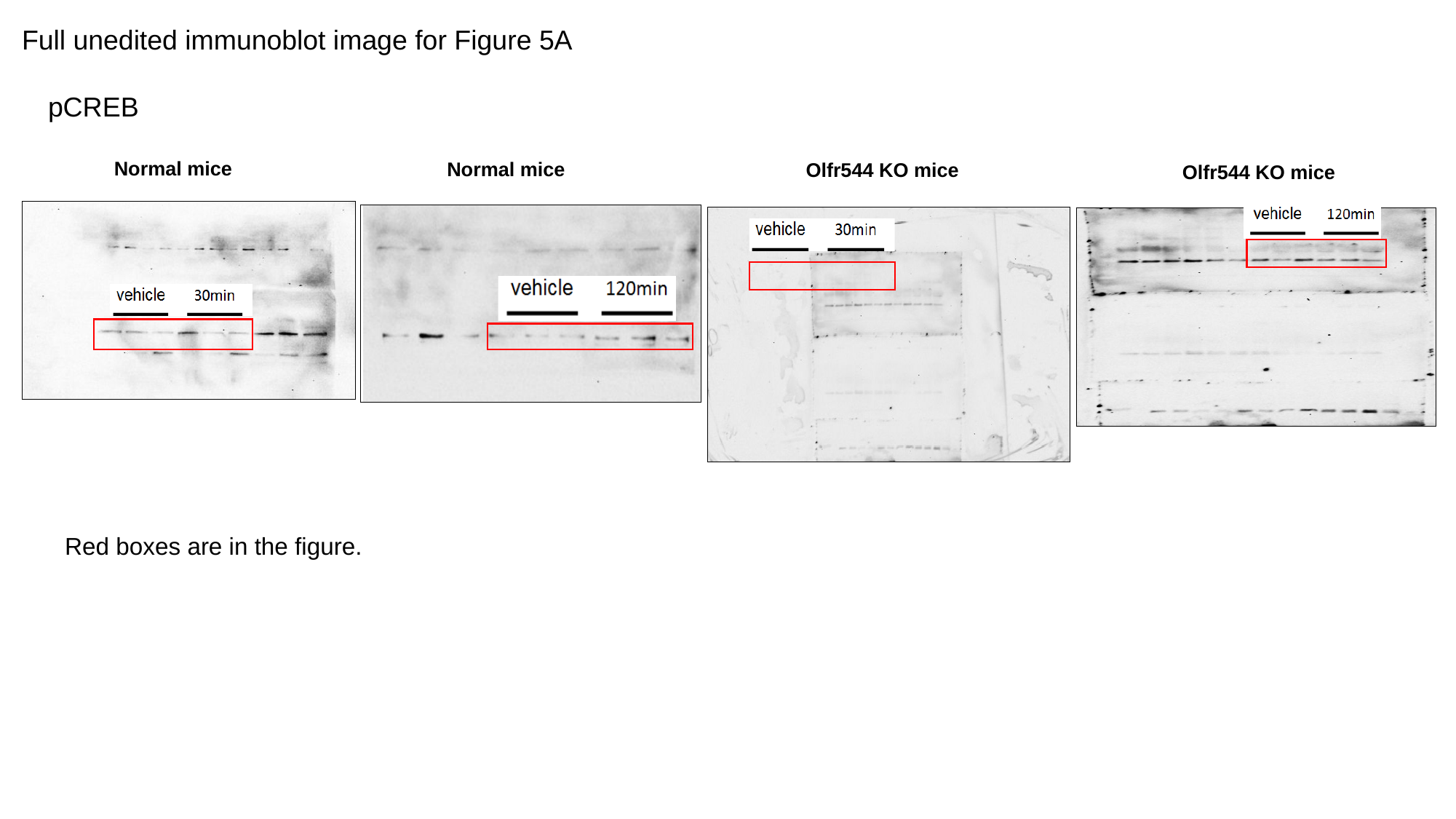

Full unedited immunoblot image for Figure 5A
pCREB
Normal mice
Normal mice
Olfr544 KO mice
Olfr544 KO mice
Red boxes are in the figure.

## Slide 7
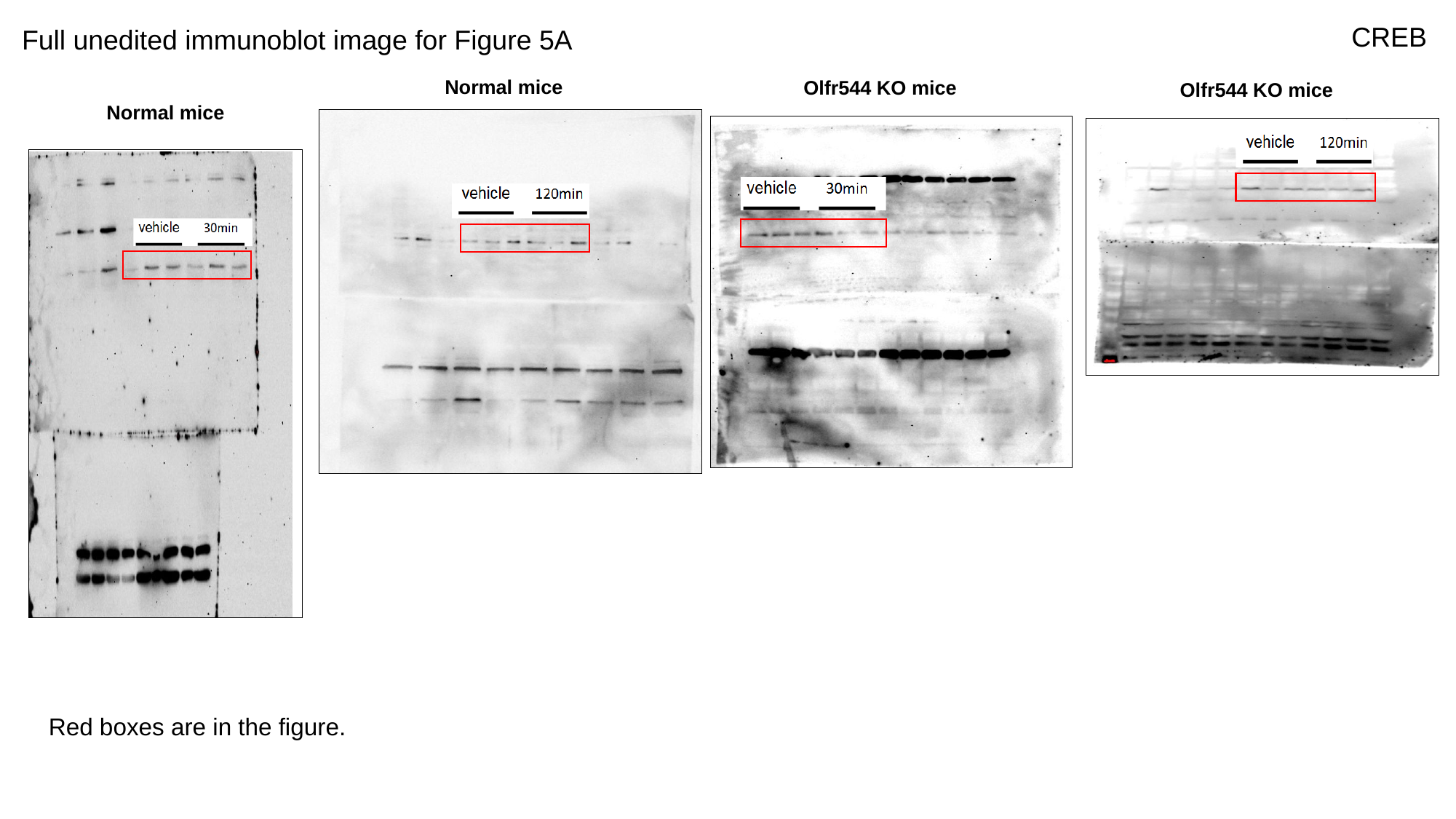

CREB
Full unedited immunoblot image for Figure 5A
Normal mice
Olfr544 KO mice
Olfr544 KO mice
Normal mice
Red boxes are in the figure.

## Slide 8
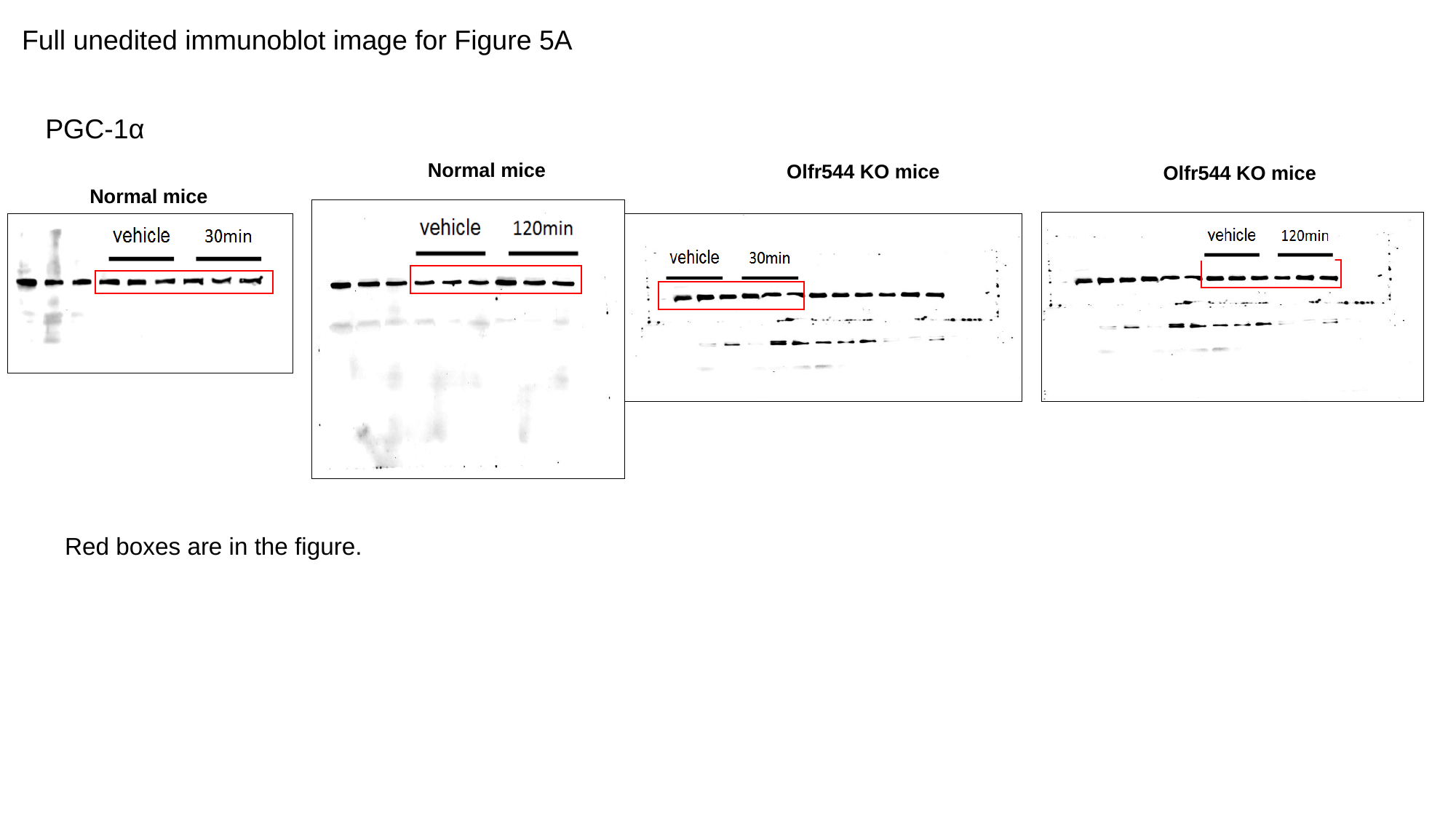

Full unedited immunoblot image for Figure 5A
PGC-1α
Normal mice
Olfr544 KO mice
Olfr544 KO mice
Normal mice
Red boxes are in the figure.

## Slide 9
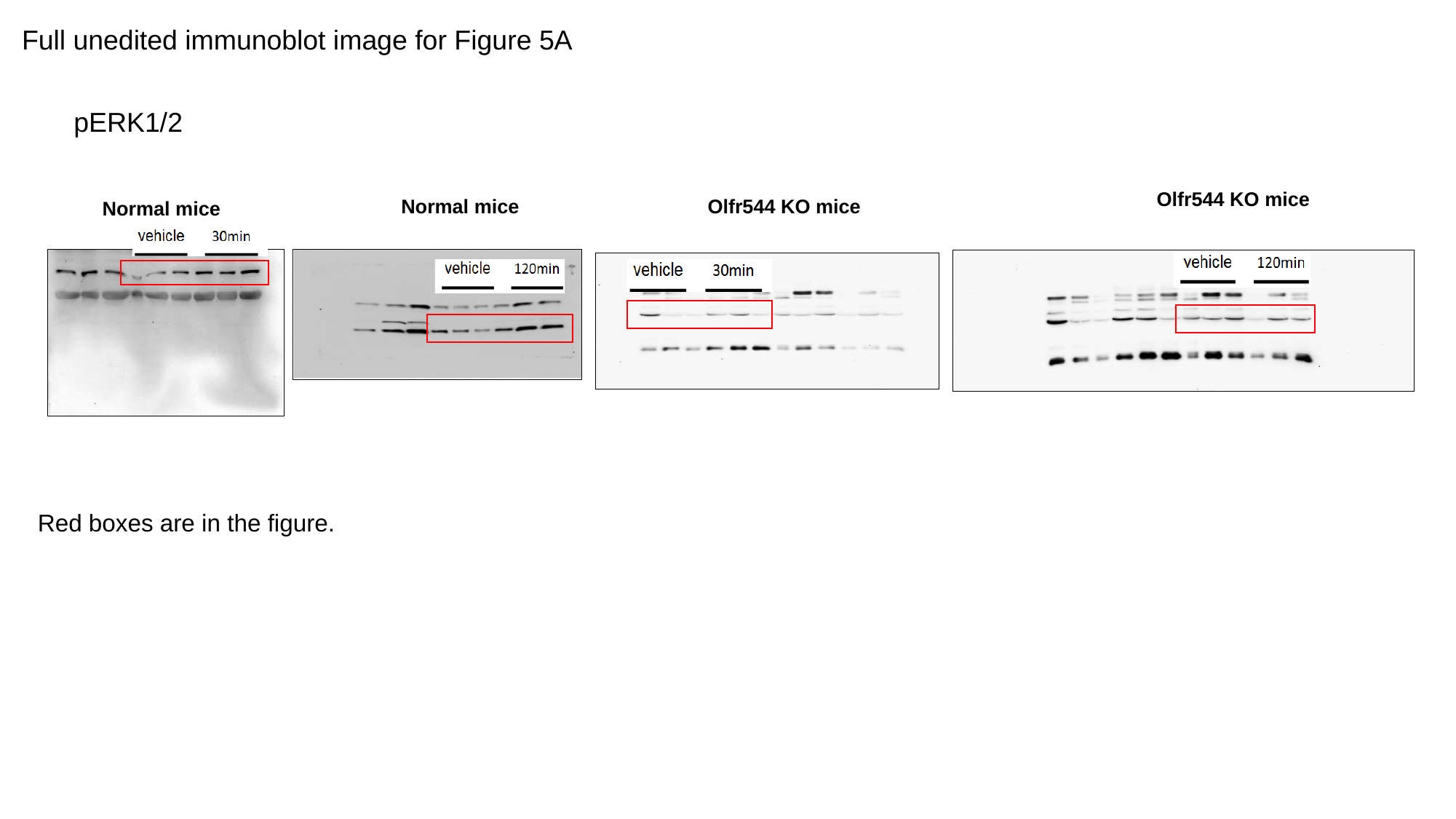

Full unedited immunoblot image for Figure 5A
pERK1/2
Olfr544 KO mice
Olfr544 KO mice
Normal mice
Normal mice
Red boxes are in the figure.

## Slide 10
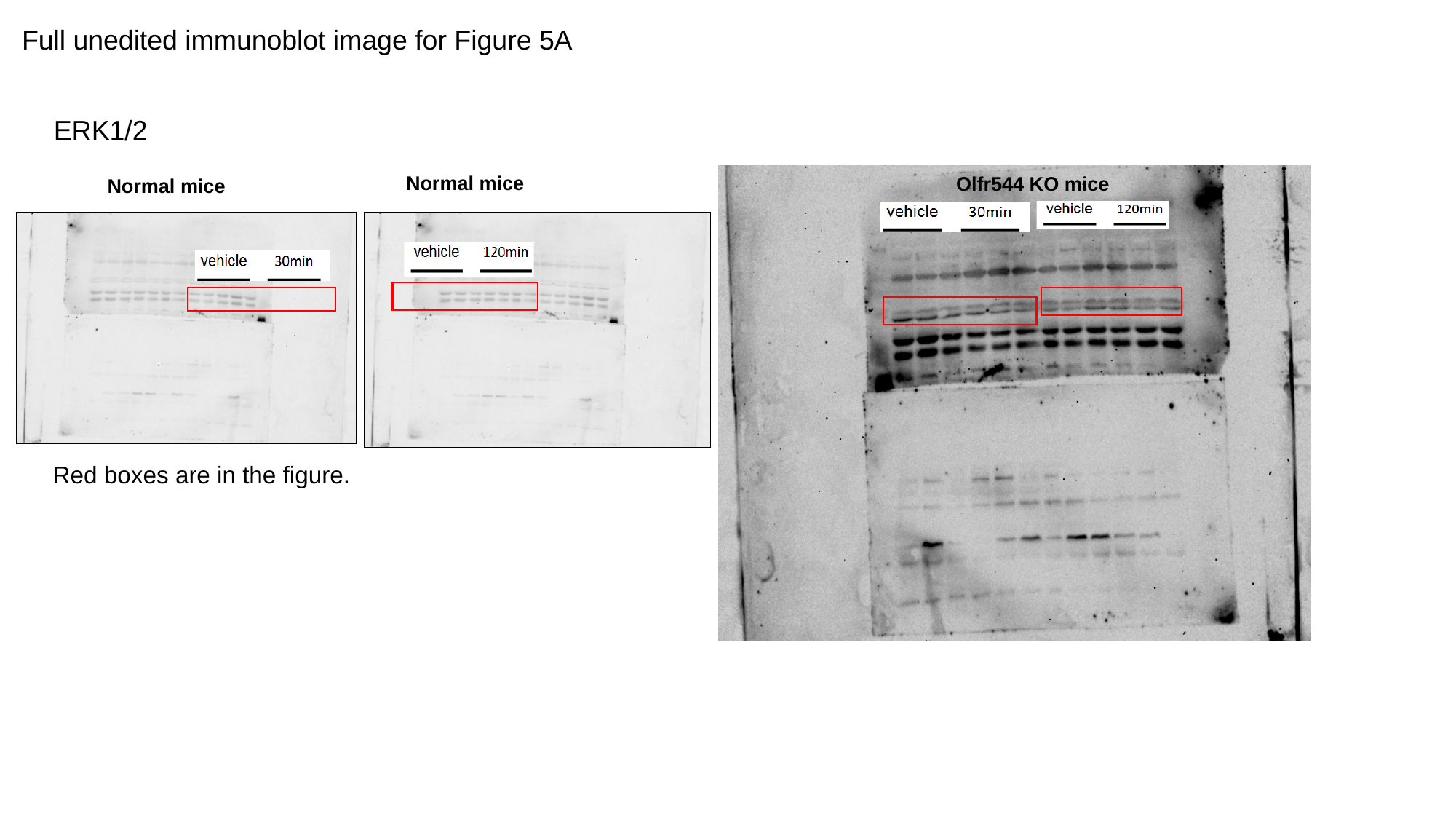

Full unedited immunoblot image for Figure 5A
ERK1/2
Normal mice
Olfr544 KO mice
Normal mice
Red boxes are in the figure.

## Slide 11
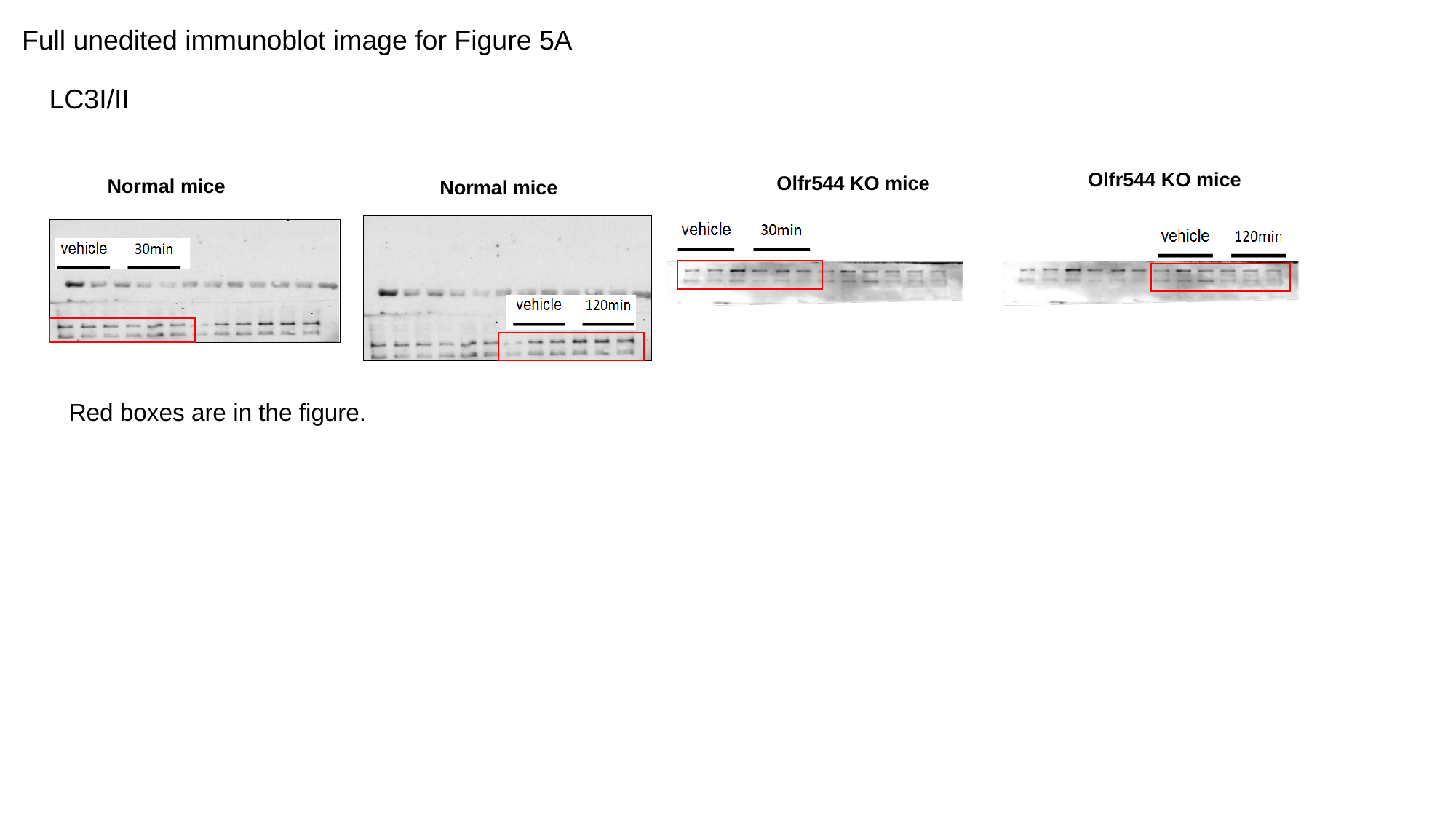

Full unedited immunoblot image for Figure 5A
LC3I/II
Olfr544 KO mice
Olfr544 KO mice
Normal mice
Normal mice
Red boxes are in the figure.

## Slide 12
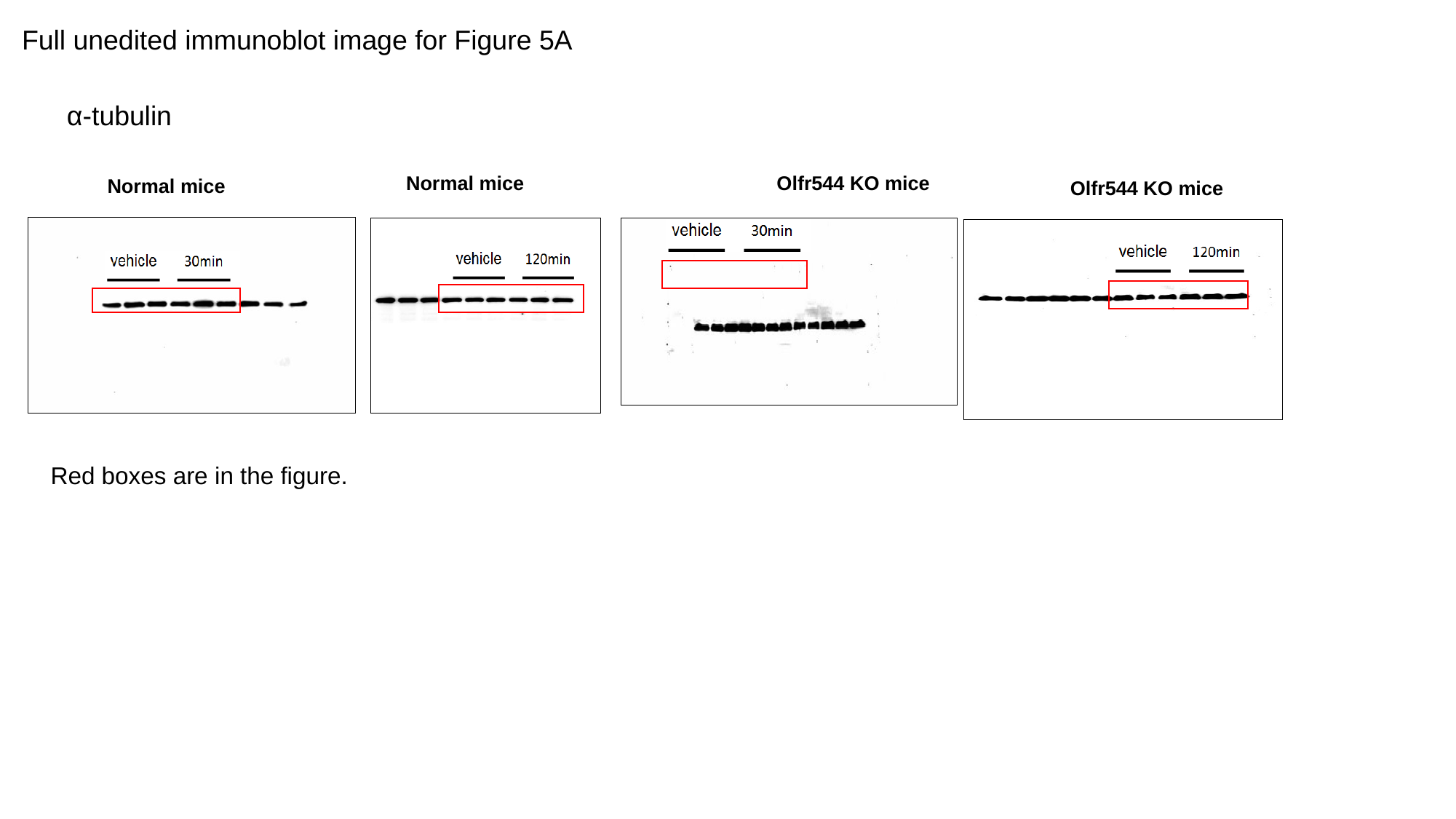

Full unedited immunoblot image for Figure 5A
α-tubulin
Olfr544 KO mice
Normal mice
Normal mice
Olfr544 KO mice
Red boxes are in the figure.
